# Host genetic factors associated with hepatocellular carcinoma in patients with hepatitis C virus infection: A systematic review

**DOI:** 10.1111/jvh.12871

**Published:** 2018-03-01

**Authors:** A. J. Walker, C. J. Peacock, V. Pedergnana, STOP‐HCV Consortium, W. L. Irving

**Affiliations:** ^1^ National Institute for Health research (NIHR) Nottingham Biomedical Research Centre Nottingham University Hospitals NHS Trust University of Nottingham Nottingham UK; ^2^ Centre for Evidence Based Medicine Department of Primary Care Health Sciences University of Oxford Oxford UK; ^3^ Wellcome Trust Centre for Human Genetics University of Oxford Oxford UK

**Keywords:** genetic predisposition, risk stratification

## Abstract

Hepatitis C virus (HCV)‐infected patients are at risk of developing hepatocellular carcinoma (HCC). Individuals at heightened risk could be targeted by intensive follow‐up surveillance. We have conducted a systematic review of the literature to identify host genetic predisposition to HCC in HCV‐infected patients. A comprehensive search of Medline and Embase databases was performed, and the strength of evidence of associations for each gene on development of HCC was evaluated. We identified 166 relevant studies, relating to 137 different genes, or combinations thereof. Seventeen genes were classified as having “good” evidence of an association, a significant association was observed for 37 genes but this finding had not yet been replicated, 56 genes had mixed or limited evidence of an association, and 27 genes showed no association. *IFNL3* */4*, *TNF‐*α and *PNPLA3* genes had the most evidence of an association. There was, however, considerable heterogeneity in study design and data quality. In conclusion, we identified a number of genes with evidence of association with HCC, but also a need for more standardized approaches to address this clinically critical question. It is important to consider the underlying mechanism of these relationships and which are confounded by the presence of other HCC risk factors and response to therapy. We also identified many genes where the evidence of association is contradictory or requires replication, as well as a number where associations have been studied but no evidence found. These findings should help to direct future studies on host genetic predisposition to HCC in HCV‐infected patients.

**Abbreviations**
ALDH2: aldehyde dehydrogenase
CAT: catalase
CSR: cirrhosis risk score
EGF: epidermal growth factor
GSTM1: glutathione S‐transferase mu 1
GSTT1: glutathione S‐transferase theta 1
GWAS: genomewide association study
HBV: hepatitis B virus
HCC: hepatocellular carcinoma
HCV: hepatitis C virus
HLA: human leucocyte antigen
HSPA1B: heat‐shock protein A1B
IFNL3: interferon lambda 3
IFNL4: interferon lambda 4
IL: interleukin
IQR: interquartile range
MDM2: mouse double minute 2 homolog
MHC: major histocompatibility complex
MICA: MHC class I polypeptide‐related sequence A
MnSOD: manganese‐dependent superoxide dismutase
PNPLA3: patatin‐like phospholipase domain‐containing protein 3
SNP: single nucleotide polymorphism
*TGF‐ß1*: transforming growth factor beta 1
TNF: tumour necrosis factor
UGT1A7: UDP glucuronosyltransferase 1 family, polypeptide A7

## 1 INTRODUCTION

Chronic hepatitis C virus (HCV) infection is one of the aetiological factors underlying the development of hepatocellular carcinoma (HCC), a commonly observed tumour whose incidence is increasing.^1^ Typically, HCC develops after sustained liver damage, where disease usually progresses from mild to severe fibrosis, then cirrhosis and eventually to HCC. Only a proportion of patients with cirrhosis will develop HCC (1%‐7% per year).^2^

The question of which HCV‐infected patients are at risk of developing HCC is an important one, especially in the era of effective direct‐acting antiviral (DAA) oral treatments.^3^ Even if most patients are likely to become free of HCV through DAA therapy, it remains important to assess which patients need continuing surveillance for early detection and treatment of HCC and other liver disease. While there are many risk factors for HCC, including alcohol use, viral hepatitis and metabolic diseases, host genetics are likely to play a crucial role. Knowledge of host genetics therefore could add discriminatory value to risk prediction tools, allowing better stratification and personalized assessment of optimal long‐term management, thereby increasing the efficacy of surveillance programmes.

This systematic literature review identified publications which examine the association between specific human genes/single nucleotide polymorphisms (SNPs) and the occurrence of HCC. We have aggregated and graded the evidence for each studied gene according to the strength of evidence for an association with HCC. As cirrhosis itself is a risk factor for HCC development, we also included reports of the association between host genetics and cirrhosis and fibrosis progression. Our aim in undertaking this task was purely utilitarian, that is with a view to developing appropriate genetic testing to aid in patient stratification and clinical management—as opposed to a mechanistic approach aiming to shed light on the molecular processes underlying the pathogenesis of HCC. We identify a large number of human genes which have been studied in relation to HCV and HCC, and classify these genes according to the strength of evidence of an association.

## 2 METHODS

### 2.1 Search strategy

We conducted a comprehensive literature search in Ovid MEDLINE and Embase databases for relevant papers, using the search strategy listed in Appendix S4, divided into the following categories: hepatocellular carcinoma, hepatitis C, genetics and risk/associations. The search strategy was developed by two researchers and checked by another independently.

To refine the search, review papers were excluded and studies were limited to the English language. Studies were limited to humans.

### 2.2 Study selection

Studies were included if they:

- Included patients infected with HCV,
- Evaluated associations between germline polymorphisms and HCC, cirrhosis or fibrosis,
- Had a case‐control design or
- Were a relevant meta‐analysis.

Study eligibility was assessed by two independent reviewers, firstly at the title level, then through abstract assessment and then by assessing the full texts. Reviewer discrepancies were resolved with the assistance of a third reviewer.

### 2.3 Data extraction

We extracted odds or hazard ratios from the selected papers, along with pertinent details of the study, including SNP reference number where available (rs#), sample size, data on adjustment or matching, the outcome studied and the comparison/control group used. Where multiple genes and/or SNPs were analysed within the same study, we extracted data for each gene/SNP. Similarly, if a study used multiple outcomes (ie HCC, cirrhosis or fibrosis), we extracted results for each of these. In cases where there were multiple comparison groups (eg HCC patients being compared with both HCV‐infected patients and healthy controls), we extracted data for the HCV‐infected comparison. Full data tables are available in Appendix S3 (here: https://figshare.com/s/2ffc9030826df2fe150e).

### 2.4 Classification of studies—strength of evidence

In order to provide an indication of the strength of evidence for each gene, all studies relating to each gene were collated together and each gene was classified into one of four categories. These categories were determined a priori and broadly were based upon genes having had a significant association which was replicated in a separate study and an absence of substantive disagreement.

Criteria for strength of evidence:

• Genes/SNPs with strong evidence
  ○ ≥2 studies with a significant association (as defined by each study) with HCC, and
  ○ Absence of substantial disagreement/negative studies or
  ○ Positive meta‐analysis;
• Studies in need of replication
  ○ Only one HCC study performed, but with a significant association found;
• Genes/SNPs with some/mixed evidence
  ○ Multiple studies carried out but with disagreement or
  ○ Positive studies, but only in cirrhosis or fibrosis, not HCC;
• Genes/SNPs with no evidence
  ○ Only negative studies or
  ○ Negative meta‐analysis (regardless of other studies).

## 3 RESULTS

### 3.1 Identified study characteristics

**Figure 1:**
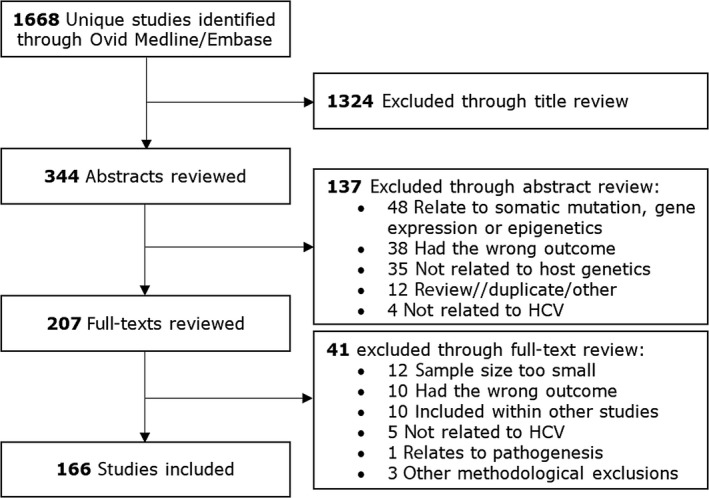
Results of the search strategy

The search criteria identified 1668 unique studies for review (Figure 1). After review, 166 studies investigating the association of host genetic polymorphisms with fibrosis progression, cirrhosis or HCC development in chronic HCV‐infected patients were selected as being relevant. One hundred and fifty‐six of these were original research articles, of which 150 were candidate gene studies, and 6 were genomewide association studies (GWAS). The remaining studies (10) were meta‐analyses. HCC was used as an outcome in 90 studies, while 45 used cirrhosis and 41 used fibrosis (some studies had multiple outcomes). The 166 selected papers are listed in Appendix S2.

There was considerable heterogeneity between studies in a number of parameters: various comparison groups were used, the most common being HCV‐infected patients without HCC, cirrhosis or fibrosis (74.7% of all studies). These comparison groups differed substantially between studies using cirrhosis/fibrosis as an outcome—which almost all compared with HCV‐infected patients (92.1%)—and HCC as an outcome, where healthy subjects (21.1%) and HCV cirrhosis patients (10.0%) were more frequently used. The median sample size in the identified research studies was 294 patients (IQR 161‐431, range 30‐6880). Studies also varied in the extent to which patients and comparators were matched, from no matching at all through to matching for age, gender, alcohol intake, source of infection and duration of infection.

A total of 133 individual genes were featured within the identified studies, as well as 4 different gene combinations, where the combined effect of polymorphisms within two or more genes, were considered. These studies may have investigated a single gene or multiple genes or in some cases multiple SNPs within the same gene.

Tables 1, 2, 3, 4 contain summarized details of all studied genes, categorized into the 4 levels of evidence described in the methods. A large amount of additional detail, including results for individual studies, hazard/odds ratios, significance, sample size and inclusion/exclusion criteria, can be found in Appendix S3 (also available here: https://figshare.com/s/2ffc9030826df2fe150e), which contains corresponding Tables 1, 2, 3, 4 with all the detail extracted from each study, for each gene. Additional detail is provided in the text below within which we have only cited examples of papers relating to HCC, and only for genes with strong evidence.

**Table 1:** Genes identified as having strong evidence of an association with HCC, in patients with HCV

Gene	HCC									Cirrhosis or fibrosis
	Candidate gene		GWAS		Meta‐analysis					
	Positive associations/number of comparisons	Number of papers	Positive associations/number of comparisons	Number of papers	Positive associations/number of comparisons	Number of papers	Total sample size	Comparison group/baseline population	Country/ethnicity	Number of papers
*ALDH2*	2/2	2	0/0	0	0/0	0	638	HCV cirrhosis, HCV infected	Japan	0
*CAT*	2/2	2	0/0	0	0/0	0	482	HCV cirrhosis, HCV infected	Caucasian, Morocco	0
*EGF*	3/3	2	0/0	0	1/1	1	578	HCV cirrhosis, HCV infected, Various	China, Egypt, Japanese	1
*GSTM1*	2/2	2	0/0	0	0/0	0	189	Chronic liver disease, HCV infected	Egypt, India	0
*GSTT1*	2/2	2	0/0	0	0/0	0	189	Chronic liver disease, HCV infected	Egypt, India	0
*HLA*	3/3	2	0/0	0	0/0	0	293	HCV infected	Spain	5
*HLA‐Bw4 + KIR3DS1*	3/3	3	0/0	0	0/0	0	776	HCV infected	Australia, Italy, Spain	0
*HSPA1B*	2/2	2	0/0	0	0/0	0	666	HCV infected, Healthy subjects	China, India	0
*IFNL3*	6/10	7	0/0	0	2/3	3	3154	Chronic HCV & HCV cirrhosis, HCV infected, HCV related HCC (age of onset)	Asian, Caucasian, Egypt, Japan, Morocco, Spain (caucasian)	6
*IL‐1b*	4/4	4	0/0	0	0/0	0	1226	HCV cirrhosis, HCV infected, Healthy subjects	Caucasian, Japan	2
*MDM2*	1/2	2	0/0	0	1/1	1	745	HCV infected, Healthy subjects	African, Asian, Caucasian, Japan, Morocco	0
*MICA*	3/5	3	1/1	1	0/0	0	15152	HCV infected	Caucasian, Japan, Spain	1
*MnSOD*	3/4	4	0/0	0	0/0	0	755	HCV cirrhosis, HCV infected	Caucasian, Egypt, Morocco	0
*PNPLA3*	2/3	3	0/0	0	1/1	1	1252	HCV cirrhosis, HCV infected, HCV related HCC (prognosis)	European, Italy, Japan, Morocco	6
*TGF‐ß1*	2/2	2	0/0	0	0/0	0	705	HCV infected	China, Egypt	5
*TNF‐a*	4/4	4	0/0	0	0/0	0	871	Chronic HCV & HCV cirrhosis, HCV cirrhosis, HCV infected, Healthy subjects	Caucasian, Chinese, Egypt	7
*UGT1A7*	5/6	5	0/0	0	0/0	0	1450	HCV infected, Healthy subjects, Viral hepatitis	China, France, German, Japan, Taiwan	1

Results for individual studies, including hazard/odds ratios, significance, sample size and inclusion/exclusion criteria can be found in Appendix S3.

**Table 2:** Genes identified as having an association with HCC in need of replication, in patients with HCV

Gene	HCC							Cirrhosis or fibrosis
	Candidate gene		GWAS					
	Positive associations/number of comparisons	Number of papers	Positive associations/number of comparisons	Number of papers	Total sample size	Comparison group/baseline population	Country/ethnicity	Number of papers
*AKR1B10*	1/1	1	0/0	0	278	HCV infected	Japan	0
*BIRC5*	1/1	1	0/0	0	568	Healthy subjects	Taiwan	0
*CCND2*	1/1	1	0/0	0	468	HCV infected	Japan	0
*CEP164*	1/1	1	0/0	0	468	HCV infected	Japan	0
*CRHR2*	1/1	1	0/0	0	376	HCV infected	Japan	0
*CYP17*	1/1	1	0/0	0	465	HCV infected	Caucasian	0
*CYP2A6*	1/1	1	0/0	0	488	Healthy subjects	Japan	0
*DEPDC5*	0/0	0	1/1	1	3312	HCV infected	Japan	0
*DHCR7*	1/1	1	0/0	0	5604	HCV infected	Caucasian, Japan	1
*DR4*	2/2	1	0/0	0	393	HCV infected	Germany	0
*GC*	1/1	1	0/0	0	5604	HCV infected	Caucasian, Japan	1
*GFRA1*	1/1	1	0/0	0	376	HCV infected	Japan	0
*GHRL*	1/4	1	0/0	0	79	HCV infected	Egypt	0
*GRP78*	1/1	1	0/0	0	468	HCV infected	Japan	0
*HSPA1L*	1/1	1	0/0	0	366	HCV infected	India	0
*hTERT*	1/1	1	0/0	0	445	Healthy subjects	China	0
*IFN‐g*	1/1	1	0/0	0	203	HCV infected	Tunisia	1
*IFN‐g+IL‐10*	1/1	1	0/0	0	203	HCV infected	Tunisia	0
*IGF‐2*	1/1	1	0/0	0	102	Healthy subjects	Taiwan	0
*IL‐10*	1/1	1	0/0	0	203	HCV infected	Tunisia	3
*IL‐17A*	1/1	1	0/0	0	75	HCV infected	Egypt	0
*IL‐18*	1/2	1	0/0	0	163	HCV infected	Tunisia	0
*IL‐23R*	2/2	1	0/0	0	292	HCV infected	Egypt	0
*KIR*	1/1	1	0/0	0	228	HCV infected	Italy	0
*KIR3DS1*	1/1	1	0/0	0	152	HCV infected	Spain	0
*mEH*	1/2	1	0/0	0	394	Healthy subjects	Italy	0
*mEPHX*	1/4	1	0/0	0	88	HCV infected	India	0
*MMP‐14*	1/1	1	0/0	0	135	Healthy subjects	Taiwan	0
*MPO*	1/1	1	0/0	0	205	HCV cirrhosis	Caucasian	0
*PTX3*	1/1	1	0/0	0	314	HCV infected	Brazil	0
*RAD23B*	1/1	1	0/0	0	468	HCV infected	Japan	0
*SCBY14*	1/1	1	0/0	0	376	HCV infected	Japan	0
*SLC22A7*	1/1	1	0/0	0	114	HCV infected	Japan	0
*SPARC*	0/0	0	1/1	1	340	Healthy subjects	Italy	0
*TLR2*	1/1	1	0/0	0	381	HCV infected	Germany	0
*TLR4*	1/2	1	0/0	0	308	HCV infected	Spain	0
*TNF‐ß*	1/1	1	0/0	0	233	HCV cirrhosis	Chinese	0

Results for individual studies, including hazard/odds ratios, significance, sample size and inclusion/exclusion criteria can be found in xAppendix S3.

**Table 3:** Genes identified as having some or mixed evidence of an association with HCC, in patients with HCV

Gene	HCC							Cirrhosis or fibrosis
	Candidate gene		Meta‐analysis					
	Positive associations/number of comparisons	Number of papers	Positive associations/number of comparisons	Number of papers	Total sample size	Comparison group/baseline population	Country/ethnicity	Number of papers
*ABCB11*	0/0	0	0/0	0				1
*AKR1C2*	0/0	0	0/0	0				1
*AKR1C3*	0/0	0	0/0	0				1
*AT‐6*	0/0	0	0/0	0				1
*BCL2L12*	0/0	0	0/0	0				1
*C4BQ40*	0/0	0	0/0	0				1
*CHI3L1*	0/0	0	0/0	0				1
*COX‐2*	0/0	0	0/0	0				1
*CPT1A*	0/0	0	0/0	0				1
*CRS7*	0/1	1	0/0	0	938	HCV infected	African American, Caucasian	2
*CSNK1A1*	0/0	0	0/0	0				1
*CTGF*	0/1	1	0/0	0	235	Chronic HCV & HCV cirrhosis	Japan	1
*CTNNB1*	0/0	0	0/0	0				1
*CXCL1*	0/0	0	0/0	0				1
*CYP19A1*	0/0	0	0/0	0				1
*CYP2C19*	1/2	2	0/0	0	712	HCV cirrhosis, Healthy subjects	Japan	0
*CYP2D6*	1/2	2	0/0	0	848	HCV infected, Healthy subjects	Caucasian, Japan	1
*CYP2E1*	1/2	2	0/0	0	530	HCV cirrhosis, HCV infected	Caucasian, Japan	0
*DDX5*	0/0	0	0/0	0				1
*ESR1*	0/0	0	0/0	0				1
*F5*	0/0	0	0/0	0				3
*FAS*	0/0	0	0/0	0				2
*FSAP*	0/0	0	0/0	0				1
*FZD4*	0/0	0	0/0	0				1
*GPx1*	1/2	2	0/0	0	482	HCV cirrhosis, HCV infected	Caucasian, Morocco	0
*HFE*	0/2	2	0/0	0	177	HCV cirrhosis, HCV infected	Caucasian, Egypt	4
*HPA*	0/0	0	0/0	0				2
*HSD17B6*	0/0	0	0/0	0				1
*HSD3B2*	0/0	0	0/0	0				1
*ICAM‐1*	0/0	0	0/0	0				1
*IFNGR2*	0/0	0	0/0	0				1
*IL‐12p40*	0/0	0	0/0	0				1
*IL‐18,IFN‐y,IL‐10(incombination)*	0/0	0	0/0	0				1
*IL‐1RA*	0/0	0	0/0	0				1
*IL‐22RA2*	0/0	0	0/0	0				1
*IRF‐3*	0/0	0	0/0	0				1
*IRF‐7*	0/0	0	0/0	0				1
*MBL‐2*	1/2	2	0/0	0	440	HCV infected, HCV/HBV coinfected	German, Italy	2
*MHC*	0/0	0	0/0	0				1
*MMP‐1*	0/0	0	0/0	0				1
*MMP‐9*	1/1	1	0/0	0	115	Healthy subjects	Egypt	1
*OAS‐1*	0/0	0	0/0	0				1
*p53*	1/1	1	0/2	2	318	HCV infected, Healthy subjects	African, Asian, Caucasian, Morocco, Various	2
*PT*	0/0	0	0/0	0				1
*RRAS*	0/0	0	0/0	0				1
*SCAF1*	0/0	0	0/0	0				1
*SCCA*	0/0	0	0/0	0				1
*SFRP2*	0/0	0	0/0	0				1
*SLC11A1*	0/0	0	0/0	0				1
*SOST*	0/0	0	0/0	0				1
*SRD5A1*	0/0	0	0/0	0				1
*TAP2*	0/0	0	0/0	0				1
*TIMP‐2*	0/0	0	0/0	0				1
*TM6SF2*	0/0	0	0/0	0				1
*VDR*	1/3	1	0/0	0	340	HCV infected	Taiwan	2
*XRCC1*	1/1	1	0/1	1	272	Healthy subjects	African, Asian, Caucasian, China	1

Results for individual studies, including hazard/odds ratios, significance, sample size and inclusion/exclusion criteria can be found in Appendix S3.

**Table 4:** Genes identified as having been studied for an association with HCC, in patients with HCV, but without any significant association found

Gene	HCC					Cirrhosis or fibrosis
	Candidate gene					
	Positive associations/number of comparisons	Number of papers	Total sample size	Comparison group/baseline population	Country/ethnicity	Number of papers
*A1AT*	0/0	0				1
*C5*	0/0	0				1
*CCR5*	0/1	1	120	HCV cirrhosis	France	0
*CD14*	0/0	0				1
*CIP2A*	0/2	1	513	Healthy subjects	China	0
*COMT*	0/1	1	465	HCV infected	Caucasian	0
*CYP1A1*	0/1	1	360	HCV infected	Caucasian	0
*CYP1A2*	0/2	2	848	HCV infected, Healthy subjects	Caucasian, Japan	0
*CYP2R1*	0/1	1	5604	HCV infected	Caucasian, Japan	1
*CYP3A4*	0/1	1	360	HCV infected	Caucasian	0
*DNMT3B*	0/1	1	278	Healthy subjects	Morocco	0
*H63D*	0/0	0				1
*HMOX1*	0/0	0				1
*IFN‐L3*	0/1	1	1118	HCV infected	Taiwan	0
*IL‐1a*	0/0	0				1
*IL‐1RN*	0/0	0				1
*IL‐4*	0/0	0				1
*MASP2*	0/1	1	263	HCV/HBV coinfected	Italy	0
*MCP‐1*	0/1	1	120	HCV cirrhosis	France	0
*miR‐146a*	0/1	1	231	HCV infected	Chinese	0
*MMP‐3*	0/0	0				1
*MTHFR*	0/0	0				1
*OPN*	0/2	1	296	HCV infected	Japanese	0
*MMP‐3*	0/0	0				1
*MTHFR*	0/0	0				1
*UGT1A1*	0/0	0				1
*YKL‐40*	0/0	0				1

Results for individual studies, including hazard/odds ratios, significance, sample size and inclusion/exclusion criteria can be found in Appendix S3.

### 3.2 Genes/SNPs with strong evidence

We identified 17 genes with strong evidence of an association with HCC in HCV‐infected patients (Table 1 and Appendix S3 available at https://figshare.com/s/2ffc9030826df2fe150e), although even within this group the level of evidence varied substantially. The number of HCC studies for each gene ranged from 2 to 7 (median 3), while for 8 of these genes, there were additional studies which found an association with cirrhosis or fibrosis. A total of 42 original studies involving HCC as an outcome made up the evidence base for these genes. An additional 25 studies involved cirrhosis or fibrosis as an outcome. A large majority of these studies used HCV‐infected patients (with or without cirrhosis) as a control group, although some (which we identify below) used healthy subjects as controls. Table 5 indicates which studies used HCV‐infected patients with cirrhosis but without HCC as their control group. Matching and adjustment were highly variable between studies, and this is best explored using the data in Appendix S3. The total number of patients studied for each gene ranged from 189 to 15 152 (median 1223), while the median sample size for each individual study (excluding meta‐analyses) was 277 (range 30‐6880).

**Table 5:** As Table 1, but restricted to studies that use patients with HCV cirrhosis as a control group

	HCC							Cirrhosisor fibrosis
	Candidate gene		GWAS					
Gene	Positive associations/number of comparisons	Number of papers	Positive associations/number of comparisons	Number of papers	Total sample size	Comparison group/baseline population	Country/ethnicity	Number of cirrhosis/fibrosis papers
*ALDH2*	1/1	1	0/0	0	170	HCV cirrhosis	Japan	1
*CAT*	1/1	1	0/0	0	205	HCV cirrhosis	Caucasian	1
*EGF*	1/1	1	0/0	0	80	HCV cirrhosis	Egypt	1
IFNL3*	1/2	1	0/0	0	438	Chronic HCV & HCV cirrhosis	Morocco	1
*IL‐1b*	1/1	1	0/0	0	253	HCV cirrhosis	Caucasian	1
*MnSOD*	0/1	1	0/0	0	205	HCV cirrhosis	Caucasian	1
*PNPLA3*	0/0	0	1/1	1	945	HCV cirrhosis	European	1
*TNF‐a*	2/2	2	0/0	0	343	Chronic HCV & HCV cirrhosis, HCV cirrhosis	Caucasian, Egypt	2

Results for individual studies, including hazard/odds ratios, significance, sample size and inclusion/exclusion criteria can be found in Appendix S3.

#### 3.2.1 ALDH2

The *ALDH2* gene encodes the mitochondrial aldehyde dehydrogenase enzyme. Two reports studied *ALDH2*, both of which used HCC as the outcome and were candidate gene studies, comprising a total of 638 patients. Kato et al^4^ found a significant association in 170 patients, with controls being age‐ and gender‐matched HCV‐infected cirrhosis patients. Alcohol was not adjusted for in this analysis but was in the second paper^5^ where a significant association was determined in 468 patients, where the comparison group was HCV‐infected patients without cirrhosis.

#### 3.2.2 CAT

The antioxidant enzyme catalase is encoded by the *CAT* gene. A total of 482 patients were studied across two different studies, both of which found an association with the SNP rs1001179.^6^, ^7^ There was a large disparity in odds ratio between these two studies (13.6 and 1.74), which may be explained in part by the different comparison groups used (noncirrhotic and cirrhotic HCV‐infected patients, respectively).

#### 3.2.3 EGF

Three original studies were identified investigating the rs4444903 SNP of the *EGF* gene, two studying HCC^8^, ^9^(578 patients in total) and one studying fibrosis, with a variety of comparison groups (including HCV‐infected and HCV‐related cirrhosis patients). The *EGF* gene encodes epidermal growth factor. All studies found a significant association with the outcome, although for one,^9^ the association was only significant for the A/A to A/G comparison (and not for A/A to G/G). Additionally, a meta‐analysis^10^ describing comparisons between HCC patients and both patients with liver disease and healthy controls found a significant overall effect.

#### 3.2.4 GSTM1

A deletion of the *GSTM1* gene—which encodes the glutathione S‐transferase mu 1 protein—was associated with HCC in two studies.^11^, ^12^ A relatively small total number of patients (n = 189) were included across these two studies, and the applicability to HCV is potentially questionable, given that only subsets of the patients in both studies were HCV‐infected, with the remaining patients having cirrhosis caused by other aetiologies.

#### 3.2.5 GSTT1

GSTT1 (encoding glutathione S‐transferase theta 1) is related to *GSTM1,* and indeed, the same studies^11^, ^12^ (and same 189 patients) which identified an association with the *GSTM1* gene deletion also found a similar association between *GSTT1* gene deletion and HCC. The same caveats regarding applicability to HCV also apply.

#### 3.2.6 HLA

While not a single gene, the *HLA* gene complex (which encodes the major histocompatibility complex) has been widely studied with respect to its association with HCC, cirrhosis and fibrosis. Three of the *HLA* genes *HLA‐Bw4,* 13 *HLA‐B18* 14 and *HLA‐DR11* 14 were reported to be associated with HCC in two studies by the same group, which investigated a total of 293 patients. An additional four studies found associations between various HLA genes/alleles and cirrhosis, including two candidate gene studies and a GWAS.

#### 3.2.7 HLA‐Bw4+KIR3DS1

In addition to the evidence of *HLA* genes associated with HCC alone, three studies^13^, ^15^, ^16^ (with a total of 776 patients) identified the pairing of *HLA‐Bw4* and *KIR3DS1* (which encodes a transmembrane glycoprotein expressed by natural killer cells) in combination as being associated with HCC.

#### 3.2.8 HSPA1B

Two studies (with a total of 666 patients) identified an association between *HSPA1B*—which encodes a heat‐shock protein—and HCC in HCV‐infected patients.^17^, ^18^ One study of 366 patients^18^ used a comparison group of HCV‐infected patients, while the other^17^ with 300 patients used healthy controls and only 44 patients in total within this study were HCV‐positive.

#### 3.2.9 IFNL3/IFNL4

The *IFNL3* (previously *IL‐28B*) and *IFNL4* genes encode the interferon lambda 3 and lambda 4 cytokines, respectively. More studies were identified for *IFNL3/IFNL4* than any other gene, with seven original studies (with a total of 3,154 patients) and three meta‐analyses using HCC as an outcome and six using cirrhosis or fibrosis. Two different SNPs were investigated—rs8099917 in 4 of the original studies and rs12979860 in 4 of them. These SNPs are in linkage disequilibrium meaning it would not be surprising if their associations with HCC are similar. For rs8099917, three of the studies found a significant association with HCC,^19^, ^20^ while one did not.^21^ However, this is not unexpected due to different patterns of correlation between SNPs in patients from different ethnic origin. For rs12979860, two studies found a significant association with HCC^22^, ^23^ and two did not.^24^, ^25^ Additionally, one study found a significant association with cirrhosis, although most studies using cirrhosis or fibrosis as an outcome did not find an association. Four of the studies were meta‐analyses, with three of these focusing on rs12979860 and HCC^26^, ^27^, ^28^ and one focusing on fibrosis. Of these, two meta‐analyses found a positive association between rs12979860 and HCC^26^, ^27^ and one found an association between fibrosis and rs12979860.

#### 3.2.10 IL‐1b

The *IL‐1b* gene encodes the cytokine interleukin‐1 beta. Four studies determined a significant association between *IL‐1b* and HCC in HCV‐infected patients,^29^, ^30^, ^31^, ^32^ whereas two studies investigated the association with cirrhosis and fibrosis and found no such association. A total of 1226 patients were included in the HCC studies, one study used healthy controls,^29^ two used HCV‐infected controls,^31^, ^33^ and the remaining one used controls with HCV‐related cirrhosis. Most of these studies also assessed or adjusted for differences in factors such as HCV genotype and alcohol use.

#### 3.2.11 MDM2

Three studies were identified for *MDM2* (mouse double minute 2 homolog, a regulator of the p53 tumour suppressor), all using HCC as an outcome,^5^, ^34^, ^35^ with a total of 745 patients. Of the two original studies, one found a positive association,^5^ the one that did not had a small sample size and used healthy subjects as the control group. The remaining study,^35^ a meta‐analysis, found a significant association in the HCV‐infected subgroup.

#### 3.2.12 MICA

There were four individual studies involving the *MICA* gene (MHC class I polypeptide‐related sequence A),^14^, ^36^, ^37^, ^38^ with a large total number of patients included (15 152, although 6880 of these were from a single meta‐analysis). The study designs are relatively homogeneous, with all using HCV‐infected patients as the control group. All studies found at least one significant association within the *MICA* gene, although some tested multiple SNPs and found associations in some but not all of the tested SNPs.^36^, ^37^ All studies used HCC as an outcome and one also found an association with cirrhosis.

#### 3.2.13 MnSOD

The *MnSOD* gene encodes the manganese‐dependent superoxide dismutase mitochondrial protein. Four relatively small studies (total 755 patients) investigated the association between HCC and *MnSOD*.^6^, ^7^, ^39^, ^40^ All studies used an HCV‐infected comparison population, and three of the studies found a significant association.^6^, ^39^, ^40^ The remaining study did not, perhaps due to difference in ethnicities between this and the other studies (Caucasian rather than North African).

#### 3.2.14 PNPLA3

Nine original studies and one meta‐analysis studied the *PNPLA3* gene (patatin‐like phospholipase domain‐containing protein 3), all investigating the same SNP, rs738409, in HCV‐infected patients. Of the three original studies using HCC as an outcome (with a total of 1252 patients),^41^, ^42^, ^43^ two of them found a positive association,^41^, ^43^ as did the meta‐analysis.^44^ The remaining six studies used cirrhosis or fibrosis as their outcome and half of these found a significant association, while three did not. This may be due to these studies being performed in a different population (Japanese) than the other studies.

#### 3.2.15 TGF‐ß1

*TGF‐ß1* is a gene encoding transforming growth factor beta 1. Six studies were identified containing 1356 patients. Of these, just two (with 705 patients)^45^, ^46^ used HCC as an outcome and both of these found a significant association using HCV‐infected controls. All but one of the studies using cirrhosis or fibrosis as an outcome found significant associations.

#### 3.2.16 TNF‐α

The *TNF‐*α gene encodes the tumour necrosis factor alpha cytokine. Ten studies studied *TNF‐*α SNPs, with four of them focusing on HCC (with 871 patients) and the same *TNF‐*α SNP (rs1800629), and all found a similar significant association, despite using a mixture of various control groups,^30^, ^46^, ^47^, ^48^ with one study (Jeng et al) using healthy controls. The association was less clear in the studies using cirrhosis and fibrosis as outcomes, where only five of nine studied associations were significant.

#### 3.2.17 UGT1A7

UDP glucuronosyltransferase 1 family, polypeptide A7, is encoded by the *UGT1A7* gene. Five of the six studies which focused on *UGT1A7* used HCC as an outcome,^33^, ^49^, ^50^, ^51^, ^52^ and all but one of these^49^ found a significant association. A total of 1450 patients made up these studies, and they used a variety of control groups including HCV‐infected patients and three studies using healthy controls. The one study which used cirrhosis as an outcome (and healthy subjects as the control) also found a positive association.

### 3.3 Studies in need of replication

We identified 37 genes which had a positive association with HCC in a single study, but with no confirmatory data from additional studies (Table 2 and Appendix S3 available at https://figshare.com/s/2ffc9030826df2fe150e). However, three of these genes (*DHCR7*,* IFN‐g*,* IL‐10*) had additional evidence of an association with cirrhosis or fibrosis. For two studies, a significant association was found for multiple SNPs within the same gene (Korner 2012 (*DR4*), Labib 2015 (*IL‐23R*)), while four studies investigated multiple SNPs and found only one significant association. The median sample size for each individual study (not including meta‐analyses) was 292 (range 75‐5604).

### 3.4 Genes/SNPs with some/mixed evidence

A total of 53 genes or gene combinations were classified as having some or mixed evidence of an association with HCC, the classification being derived from a number of reasons (Table 3 and Appendix S3 available at https://figshare.com/s/2ffc9030826df2fe150e). For example some genes, such as *FAS,* had only studies relating to cirrhosis and/or fibrosis. Others, such as *CYP2E1,* have one study finding a positive association with HCC, but then others which failed to replicate this association. The total number of patients studied for each gene ranged from 30 to 6472 (median 1223), while the median sample size for each individual study (not including meta‐analyses) was 296 (range 30‐6472).

### 3.5 Genes/SNPs with only negative studies

There were 27 genes identified where none of the studies found a significant association between the gene and liver outcomes (Table 4 and Appendix S3 available at https://figshare.com/s/2ffc9030826df2fe150e). All but two of the genes (*CYP1A2* and *CYP2R1*) had just one study associated with them, although some looked at multiple SNPs within the same gene. Seventeen of the studies investigated the association with HCC and 13 with cirrhosis or fibrosis. The median sample size for each individual study (not including meta‐analyses) was 262 (range 61‐5604).

## 4 DISCUSSION

The most striking features of this review are firstly the wide breadth, but disappointingly shallow depth, of the literature on what should be a critical area of research, *viz*. how can studies of host genetics improve stratification and management of patients infected with HCV who are at risk of developing HCC. From a search which yielded 1668 publications, we identified 166 relevant studies measuring associations with HCC, cirrhosis or fibrosis in HCV‐infected patients in 137 different genes. Of these, we determined 16 genes and one combination of genes with at least some validated evidence of an association with HCC, according to a priori criteria, but a large number of other genes where the evidence is currently insufficient to allow conclusions to be drawn; and secondly, the enormous heterogeneity in study design and data quality across the different studies reported in the literature. There was a large variation in study size, although this did not appear to vary substantially according to whether the genes had a high or low level of evidence. Many of the larger studies were GWA studies, which of necessity require a larger sample size due to the need for multiple tests for significance. There is evidently no agreement on what constitutes an appropriate control group against which to compare patients with HCV‐associated HCC with most studies using HCV‐infected patients, a smaller number HCV‐infected patients with cirrhosis and a small minority using healthy subjects. Our view is that HCV‐infected patients with cirrhosis but without HCC are the most appropriate control group, and therefore, we have highlighted studies of those genes/SNPs with the strongest evidence of an association which used that particular control group in Table 5. There was also substantial variation in the degree of adjustment for confounding factors. Age and sex adjustment was common, and alcohol intake was also used (although it is difficult to collect reliable alcohol data). Relatively few studies were able to adjust for duration of infection, a critical potential confounding factor relevant to a number of genes. Many studies tested several different genes, or SNPs within genes, and found associations in some of them. While some studies appropriately adjusted for use of multiple comparisons, others did not. Some studies focussed solely on HCV‐associated HCC, while in others, the HCV‐infected patients were only a subset of the case group. HBV and HCV–co‐infected patients also pose a methodological problem. Such studies should be interpreted with more caution, as the associations of SNPs with HCC may also be mediated through HBV infection.

The criteria for assessment of strength of association of individual genes/SNPs used in this review are not especially sophisticated, being based on the common‐sense approach of whether or not individual associations have been independently replicated. The scope and quality of the literature on this subject is mostly so variable that it is not possible to apply any more formal approaches, such as meta‐analysis, to evaluate the strength of associations. Our approach has, nevertheless, highlighted a number of genes/SNPs that will merit further analysis in better designed, larger‐scale studies addressing this clinically relevant issue.

There is a clinical need to identify patients who are at increased risk of HCC in a population of HCV‐infected patients. Given this, it is conceivable that the genes identified as having strong evidence of an association could, with appropriate further investigation and validation, be combined into a genetic risk score for HCC in HCV‐infected patients. The cirrhosis risk score 7 (CRS7) aims to do this for cirrhosis and fibrosis,^53^, ^54^ although we did not find any significant associations between CRS7 and HCC in our search. Combining a putative HCC risk score with other clinical data could create a composite score with a powerful ability to stratify and predict risk, and thereby impact on patient care pathways.

We also identified 37 genes, which had some evidence of an association, but without replication in an independent study. These genes therefore have potential—but not yet appropriately determined—utility in a composite score as described above. The 53 genes categorized into the some/mixed evidence group are perhaps the most heterogeneous in terms of the level of evidence. Some in this category only had evidence of association with cirrhosis or fibrosis, which may indicate some predisposition to HCC, but further such investigation of the direct association is required to conclude this with certainty. Other genes had studies with contradictory conclusions, meaning their potential utility as genes defining risk is uncertain. For the 27 genes without any apparent evidence of association with HCC, inclusion on this list does not necessarily rule out the possibility of an association. Equally, it is likely that other genes have been studied without finding an association but these data remain unpublished.

Due to the number of genes featured in this review, we did not attempt to measure the extent of publication bias within the evidence for each gene. It is therefore difficult to know to what extent unpublished contradictory evidence might have altered the classifications made.

Equally, we did not aim to determine any definitive causality between the genes identified in this study and the liver disease outcomes, taking a utilitarian, not mechanistic approach to the data. Some of the described genetic associations may arise from direct effects of the SNPs on some aspect of carcinogenesis. However, others may arise in the context of potential confounding effects, with potential mechanisms other than direct induction of HCC. For example, genes such as *IFNL3* and *IFNL4* are known to be associated with treatment response in those treated with interferon regimens.^55^ For these genes, therefore, the causal mechanism for the association with HCC incidence may be related to the duration of infection, rather than any direct effect on liver disease progression or hepatocarcinogenesis. The *IFNL3/IFNL4* studies featured here were broadly not able to adjust for this duration of infection. In the new era of DAA treatments, it is possible that these associations will cease to exist as response to interferon therapy becomes less relevant. Another example of potential confounding is *PNPLA3*, which has previously been linked to alcoholic liver disease.^56^ Although some studies adjusted for alcohol consumption, it is almost certain that at least some of the mechanism of association with HCV is due to confounding by alcoholic liver disease. These alternative mechanisms do not necessarily detract from the gene's utility as a predictor of HCC, but must be carefully considered in any modelling.

We included the evidence surrounding cirrhosis and fibrosis within this review as the typical disease course tends to progress from fibrosis to cirrhosis and eventually HCC. However, only a small percentage of patients who have cirrhosis are likely to get HCC (1%‐7% per year).^2^ This means that genes associated with cirrhosis and fibrosis do not necessarily translate to risk of HCC as well. It may be that different types of genes, such as those involved in cell cycle regulation, are more likely to be associated with HCC, while other genes are likely to be involved in the pathogenesis of fibrosis and cirrhosis.

It is crucial that evidence for each gene should be assessed in detail before drawing conclusions as to their utility for risk stratification. This systematic review of the evidence surrounding the link between host genetic factors and HCC has produced a significant aggregation of the evidence available within the literature addressing this topic. We have identified a number of methodological difficulties in studies relating to this issue and the need for a more rigorous and systematic approach to identification and validation of candidate genes. We have also identified a number of host SNPs with at least some validated evidence of an association with HCC in HCV‐infected patients, which we hope will act as a springboard to further studies and the ultimate identification of a clinically useful host genetic signature enabling stratification of individual patient risk of HCC development.

## CONFLICT OF INTEREST

AJ Walker, CJ Peacock and V Pedergnana declared no conflict of interests. WL Irving has received research grants from GSK, Pfizer, Abbvie, Bristol‐Myers Squibb, Gilead Sciences and Janssen‐Cilag; has acted on advisory boards for Novartis, MSD and Bristol‐Myers Squibb; has received speaker fees from Janssen‐Cilag, Roche Diagnostics and Gilead Sciences; and has received educational grants from Boehringer‐Ingelheim and Gilead Sciences.

## AUTHORS' CONTRIBUTIONS

WLI and AJW conceived the review. CJP and AJW performed the literature review. All 4 named authors performed data analysis, constructed and critically reviewed the manuscript. STOP‐HCV provided the funding for the study.
