# Supplementary material for: Host genetic factors associated with hepatocellular carcinoma in patients with hepatitis C virus infection: A systematic review
Source: J Viral Hepat. 2018 Mar 1;25(5):442–56. doi: 10.1111/jvh.12871 (PMC6321980; doi:10.1111/jvh.12871)
Supplement: Supplementary file 1 [file JVH-25-442-s001.docx]

Appendix A

STOP-HCV consortium members

Eleanor Barnes^1^, Jonathan Ball^2^, Gary Burgess^3^, Graham Cooke^4^, John Dillon^5^, Charles Gore^6^, Graham Foster^7^, Neil Guha^2^, Rachel Halford^6^, Cham Herath^8^, Chris Holmes^9^, Anita Howe^10^, Emma Hudson^1^, William Irving^2^, Salim Khakoo^11^, Diana Koletzki^12^, Natasha Martin^13^, Tamyo Mbisa^14^, Jane McKeating^15^, John McLauchlan^16^, Alec Miners^17^, Andrea Murray^18^, Peter Shaw^19^, Peter Simmonds^1^, Chris Spencer^20^, Paul Targett-Adams^21^, Emma Thomson^16^, Peter Vickerman^22^ & Nicole Zitzmann^36^

1 University of Oxford, Peter Medawar Building for Pathogen Research, South Parks Road, Oxford OX1 3SY, UK;

2 University of Nottingham, Queen’s Medical Centre, Nottingham, NG7 2UH;

3 Conatus Pharmaceuticals, 16745 West Bernardo Drive, Suite 200, San Diego, California 92127, USA;

4 Imperial College London, Wright Fleming Institute, London, UK;

5 University of Dundee, Ninewells Hospital & Medical School, Dundee, DD1 9SY, UK;

6 Hepatitis C Trust, 27 Crosby Row, London SE1 3YD, UK;

7 Queen Mary’s University of London, 4 Newark Street, London E1 4AT, UK;

8 Gilead Sciences, Stockley Park, 2 Roundwood Avenue, Middlesex UB11 1AF, UK;

9 University of Oxford, 24–29 St Giles’, Oxford OX1 3LB, UK;

10 BC Centre for Excellence in HIV/AIDS, St Paul’s Hospital, 608–1081 Burrard Street, Vancouver, British Columbia, Canada V6Z 1Y6;

11 University of Southampton, University Road, Southampton, SO17 1BJ, UK;

12 Janssen Diagnostics, Turnhoutseweg, 30, 2340 Beerse, Belgium;

13 UC San Diego, La Jolla, California 92093-0507, USA;

14 Public Health England, 61 Colindale Avenue, London NW9 5EQ, UK;

15 University of Birmingham, Centre for Human Virology, Edgbaston, Birmingham, B15 2TT, UK;

16 University of Glasgow, MRC-CVR, 464 Bearsden Road, Glasgow, G61 1QH, UK;

17 London School of Hygiene & Tropical Medicine, 15–17 Tavistock Place, London, WC1H 9SH, UK;

18 OncImmune Limited, Clinical Sciences Building, Nottingham City Hospital, Hucknall Road, Nottingham NG5 1PB, UK;

19 Merck & Co., Inc., Kenilworth, New Jersey 07033, USA;

20 University of Oxford, Wellcome Trust Centre for Human Genetics, Roosevelt Drive, Oxford OX3 7BN, UK;

21 Medivir AB, Box 1086, 141 22 Huddinge, Sweden;

22 University of Bristol, Oakfield House, Oakfield Grove, Clifton BS8 2BN, UK;

23 University of Oxford, South Parks Road, Oxford OX1 3QU, UK.
