# Supplementary material for: Host genetic factors associated with hepatocellular carcinoma in patients with hepatitis C virus infection: A systematic review
Source: J Viral Hepat. 2018 Mar 1;25(5):442–56. doi: 10.1111/jvh.12871 (PMC6321980; doi:10.1111/jvh.12871)
Supplement: Supplementary file 2 [file JVH-25-442-s002.docx]

Appendix B

Papers selected within the systematic review

Abbas E, Shaker O, Abd El Aziz G, Ramadan H, Esmat G. Epidermal growth factor gene polymorphism 61A/G in patients with chronic liver disease for early detection of hepatocellular carcinoma: a pilot study. Eur J Gastroenterol Hepatol [Internet]. England: Abbas,Emad. Faculty of Medicine, Department of Medical Biochemistry and Molecular Biology, Cairo University, Cairo, Egypt.; 2012;24(4):458–63. Available from: http://ovidsp.ovid.com/ovidweb.cgi?T=JS&PAGE=reference&D=medl&NEWS=N&AN=22293333

Abd El-Moneim E, Younis FA, Allam N, Gameel K, Osman M. Gene deletion of glutathione S-transferase M1 and T1 and risk factors of hepatocellular carcinoma in Egyptian patients. Egypt J Immunol [Internet]. Egypt: Abd El-Moneim,Elhamy. Department of Clinical Pathology, National Liver Institute, Menoufiya University, Egypt.; 2008;15(2):125–34. Available from: http://ovidsp.ovid.com/ovidweb.cgi?T=JS&PAGE=reference&D=med5&NEWS=N&AN=20306695

Agúndez JA, García-Martin E, Maestro ML, Cuenca F, Martínez C, Ortega L, et al. Relation of IL28b gene polymorphism with biochemical and histological features in hepatitis C virus-induced liver disease. Waris G, editor. PLoS One [Internet]. J. M. Ladero, Liver Unit, Service of Gastroenterology, Hospital Clinico San Carlos, Department of Medicine, Medical School, Universidad Complutense, Instituto de Investigacion Sanitaria del Hospital Clinico San Carlos (IdISSC), Madrid, Spain. E-mail: jlad: Public Library of Science (185 Berry Street, Suite 1300, San Francisco CA 94107, United States); 2012 May 29 [cited 2016 Dec 7];7(5):no pagination. Available from: http://dx.plos.org/10.1371/journal.pone.0037998

Agundez JA, Garcia-Martin E, Devesa MJ, Carballo M, Martinez C, Lee-Brunner A, et al. Polymorphism of the TLR4 gene reduces the risk of hepatitis C virus-induced hepatocellular carcinoma. Oncology [Internet]. Switzerland: Agundez,Jose A. Department of Pharmacology, Medical School, University of Extremadura, Badajoz, Spain.; 2012;82(1):35–40. Available from: http://ovidsp.ovid.com/ovidweb.cgi?T=JS&PAGE=reference&D=medl&NEWS=N&AN=22286521

Aikawa T, Kojima M, Onishi H, Tamura R, Fukuda S, Suzuki T, et al. HLA DRB1 and DQB1 alleles and haplotypes influencing the progression of hepatitis C. J Med Virol [Internet]. UNITED STATES: Aikawa,T. Aikawa Internal Hospital, Japan.; 1996;49(4):274–8. Available from: http://ovidsp.ovid.com/ovidweb.cgi?T=JS&PAGE=reference&D=med4&NEWS=N&AN=8877758

Akuta N, Chayama K, Suzuki F, Someya T, Kobayashi M, Tsubota A, et al. Risk factors of hepatitis C virus-related liver cirrhosis in young adults: Positive family history of liver disease and transporter associated with antigen processing 2 (TAP2) *0201 allele. J Med Virol [Internet]. N. Akuta, Department of Gastroenterology, Toranomon Hospital, 2-2-2 Toranomon, Minato-ku, Tokyo 105-0001, Japan. E-mail: akuta-gi@umin.ac.jp: Akuta,N. Division of Gastroenterology, Toranomon Hospital, 2-2-2 Toranomon, Minato-ku, Tokyo, 105-0001, Japan. akuta-gi@umin.ac.jp; 2001;64(2):109–16. Available from: http://ovidsp.ovid.com/ovidweb.cgi?T=JS&PAGE=reference&D=emed5&NEWS=N&AN=2001165922

Almeida Pereira Leite ST, Marques-Guimaraes N, Silva-Oliveira JC, Dutra-Souto FJ, Alves-dos-Santos R, Bassi-Branco CL. The X-ray repair cross complementing protein 1 (XRCC1) rs25487 polymorphism and susceptibility to cirrhosis in Brazilian patients with chronic viral hepatitis. Ann Hepatol [Internet]. Mexico: Fundacion Clinica Medica Sur (Puente de Piedra 150, Toriello Guerra, C.P. 14050, Mexico); 2013;12(5):733–9. Available from: http://ovidsp.ovid.com/ovidweb.cgi?T=JS&PAGE=reference&D=medl&NEWS=N&AN=24018491

Alves Pedroso ML, Boldt ABW, Pereira-Ferrari L, Steffensen R, Strauss E, Jensenius JC, et al. Mannan-binding lectin MBL2 gene polymorphism in chronic hepatitis C: association with the severity of liver fibrosis and response to interferon therapy. Clin Exp Immunol [Internet]. England: Alves Pedroso,M L. Department of Internal Medicine, Hospital de Clinicas, Curitiba, Brazil.; 2008;152(2):258–64. Available from: http://ovidsp.ovid.com/ovidweb.cgi?T=JS&PAGE=reference&D=med5&NEWS=N&AN=18336595

Asahina Y, Tsuchiya K, Nishimura T, Muraoka M, Suzuki Y, Tamaki N, et al. Genetic variation near interleukin 28B and the risk of hepatocellular carcinoma in patients with chronic hepatitis C. J Gastroenterol [Internet]. Japan: Asahina,Yasuhiro. Department of Gastroenterology and Hepatology, Musashino Red Cross Hospital, 1-26-1 Kyonan-cho, Musashino-shi, Tokyo, 180-8610, Japan, asahina.gast@tmd.ac.jp.; 2014;49(7):1152–62. Available from: http://ovidsp.ovid.com/ovidweb.cgi?T=JS&PAGE=reference&D=medl&NEWS=N&AN=23860735

Asim M, Khan LA, Husain SA, Husain S, Sarma MP, Ahmad I, et al. Genetic polymorphism of glutathione S transferases M1 and T1 in Indian patients with hepatocellular carcinoma. Dis Markers [Internet]. Netherlands: Asim,Mohammad. Department of Medicine, Maulana Azad Medical College, University of Delhi, India.; 2010;28(6):369–76. Available from: http://ovidsp.ovid.com/ovidweb.cgi?T=JS&PAGE=reference&D=med5&NEWS=N&AN=20683151

Bagir G.S., Oksuz M, Dogan U.B., Egesel T. Relationship between hemochromatosis gene mutations and degree of fibrosis in liver disease associated with chronic hepatitis B and C [Internet]. Turkiye Klinikleri Journal of Medical Sciences. U. B. Dogan, Clinics of Gastroenterology, Adana Numune Training and Research Hospital, Adana, Turkey. E-mail: ubdogan@hotmail.com: Turkiye Klinikleri Journal of Medical Sciences (Talapapa Bulvary no. 102, Hamammonu 1 06230, Turkey); 2012. p. 917–24. Available from: http://tipbilimleri.turkiyeklinikleri.com/download_pdf.php?id=62701

Bahr MJ, el Menuawy M, Boeker KHW, Musholt PB, Manns MP, Lichtinghagen R. Cytokine gene polymorphisms and the susceptibility to liver cirrhosis in patients with chronic hepatitis C. Liver Int [Internet]. England: Bahr,Matthias J. Department of Gastroenterology, Hepatology and Endocrinology, Medizinische Hochschule Hannover, Hannover, Germany.; 2003;23(6):420–5. Available from: http://ovidsp.ovid.com/ovidweb.cgi?T=JS&PAGE=reference&D=med4&NEWS=N&AN=14986816

Baur K, Mertens JC, Schmitt J, Iwata R, Stieger B, Eloranta JJ, et al. Combined effect of 25-OH vitamin D plasma levels and genetic vitamin D receptor (NR 1I1) variants on fibrosis progression rate in HCV patients. Liver Int [Internet]. England: Baur,Katharina. Division of Gastroenterology & Hepatology, University Hospital Zurich (USZ), Zurich, Switzerland.; 2012;32(4):635–43. Available from: http://ovidsp.ovid.com/ovidweb.cgi?T=JS&PAGE=reference&D=medl&NEWS=N&AN=22151003

Berres M-L, Papen S, Pauels K, Schmitz P, Zaldivar MM, Hellerbrand C, et al. A functional variation in CHI3L1 is associated with severity of liver fibrosis and YKL-40 serum levels in chronic hepatitis C infection. J Hepatol [Internet]. England: Berres,Marie-Luise. Department of Internal Medicine III, University Hospital Aachen, RWTH Aachen, Pauwelsstr. 30, D-52057 Aachen, Germany.; 2009;50(2):370–6. Available from: http://ovidsp.ovid.com/ovidweb.cgi?T=JS&PAGE=reference&D=med5&NEWS=N&AN=19070929

Bouzgarrou N, Hassen E, Schvoerer E, Stoll-Keller F, Bahri O, Gabbouj S, et al. Association of interleukin-18 polymorphisms and plasma level with the outcome of chronic HCV infection. J Med Virol [Internet]. United States: Bouzgarrou,N. Laboratory of Molecular Immuno-oncology, Faculty of Medicine, Monastir, Tunisia. nadia.bouzgarrou@lycos.com; 2008 Apr [cited 2016 Dec 7];80(4):607–14. Available from: http://ovidsp.ovid.com/ovidweb.cgi?T=JS&PAGE=reference&D=med5&NEWS=N&AN=18297714

Bouzgarrou N, Hassen E, Gabbouj S, Schvoerer E, Ben Mami N, Triki H, et al. Lack of effect of tumor necrosis factor-alpha -308 G/A polymorphism on severity of liver fibrosis in Tunisian hepatitis C virus (HCV)-infected patients. Gastroenterol Clin Biol [Internet]. France: Bouzgarrou,N. Molecular Immuno-Oncology Laboratory, Faculty of Medicine, 5019 Monastir, Tunisia. nadia.bouzgarrou@lycos.com; 2010;34(4-5):297–304. Available from: http://ovidsp.ovid.com/ovidweb.cgi?T=JS&PAGE=reference&D=med5&NEWS=N&AN=20537485

Bouzgarrou N, Hassen E, Bahri O, Gabbouj S, Mami N Ben, Triki H, et al. Combined effect of pro- and anti-inflammatory cytokine gene polymorphisms on susceptibility to liver cirrhosis in Tunisian HCV-infected patients [Internet]. Hepatology International N. Bouzgarrou, Molecular Immuno-oncology Laboratory, Faculty of Medicine, Monastir, Tunisia. E-mail: nadia.bouzgarrou@lycos.com: Springer New York (233 Springer Street, New York NY 10013-1578, United States); Jun, 2011 p. 681–7. Available from: http://www.ncbi.nlm.nih.gov/pubmed/21484147

Bouzgarrou N, Hassen E, Farhat K, Bahri O, Gabbouj S, Maamouri N, et al. Combined analysis of interferon-gamma and interleukin-10 gene polymorphisms and chronic hepatitis C severity. Hum Immunol [Internet]. United States: Bouzgarrou,Nadia. Molecular Immuno-oncology Laboratory, Faculty of Medicine, Monastir, Tunisia.; 2009;70(4):230–6. Available from: http://ovidsp.ovid.com/ovidweb.cgi?T=JS&PAGE=reference&D=med5&NEWS=N&AN=19480854

Cangussu LOF, Teixeira R, Campos EF, Rampim GF, Mingoti SA, Martins-Filho OA, et al. HLA class II alleles and chronic hepatitis C virus infection. Scand J Immunol [Internet]. England: Cangussu,L O F. Viral Hepatitis Division, Instituto Alfa de Gastroenterologia, Hospital das Clinicas/UFMG, Belo Horizonte, Minas Gerais, Brazil.; 2011;74(3):282–7. Available from: http://ovidsp.ovid.com/ovidweb.cgi?T=JS&PAGE=reference&D=med5&NEWS=N&AN=21535077

Carmo RF, Aroucha D, Vasconcelos LRS, Pereira LMMB, Moura P. Genetic variation in PTX3 and plasma levels associated with hepatocellular carcinoma in patients with HCV [Internet]. Journal of Viral Hepatitis. R.F. Carmo, Colegiado de Ciencias Farmaceuticas, Universidade Federal Do Vale Do Sao Francisco (UNIVASF), Av. Jose de Sa Manicoba, s/n, Centro, Petrolina, PE CEP: 56304-917, Brazil. E-mail: rodrigo.carmo@univasf.edu.br: Blackwell Publishing Ltd; 2016. p. 116–22. Available from: http://www.blackwell-science.com/jvh

Chang K-C, Tseng P-L, Wu Y-Y, Hung H-C, Huang C-M, Lu S-N, et al. A polymorphism in interferon L3 is an independent risk factor for development of hepatocellular carcinoma after treatment of hepatitis C virus infection. Clin Gastroenterol Hepatol [Internet]. United States: Chang,Kuo-Chin. Division of Hepato-Gastroenterology, Department of Internal Medicine, Kaohsiung Chang Gung Memorial Hospital, Chang Gung University College of Medicine, Kaohsiung, Taiwan.; 2015;13(5):1017–24. Available from: http://ovidsp.ovid.com/ovidweb.cgi?T=JS&PAGE=reference&D=medl&NEWS=N&AN=25460552

Chau TK, Marakami S, Kawai B, Nasu K, Kubota T, Ohnishi A. Genotype analysis of the CYP2C19 gene in HCV-seropositive patients with cirrhosis and hepatocellular carcinoma. Life Sci [Internet]. ENGLAND: Chau,T K. Department of Internal Medicine, Daisan Hospital, Jikei University School of Medicine, Komae, Tokyo, Japan.; 2000;67(14):1719–24. Available from: http://ovidsp.ovid.com/ovidweb.cgi?T=JS&PAGE=reference&D=med4&NEWS=N&AN=11021356

Chen T-Y, Li Y-C, Liu Y-F, Tsai C-M, Hsieh Y-H, Lin C-W, et al. Role of MMP14 gene polymorphisms in susceptibility and pathological development to hepatocellular carcinoma. Ann Surg Oncol [Internet]. United States: Chen,Tzy-Yen. School of Medicine, Chung Shan Medical University, Taichung, Taiwan.; 2011;18(8):2348–56. Available from: http://ovidsp.ovid.com/ovidweb.cgi?T=JS&PAGE=reference&D=med5&NEWS=N&AN=21298348

Cong N, Chen H, Bu W-Z, Li J-P, Liu N, Song J-L. miR-146a G>C polymorphisms and risk of hepatocellular carcinoma in a Chinese population. Tumour Biol [Internet]. Netherlands: Cong,Ning. Department of Surgical Oncology (Interventional Therapy), Shandong Tumor Hospital and Institute, Jinan, 250117, China.; 2014;35(6):5669–73. Available from: http://ovidsp.ovid.com/ovidweb.cgi?T=JS&PAGE=reference&D=medl&NEWS=N&AN=24615520

Corchado S, Marquez M, Montes de Oca M, Romero-Cores P, Fernandez-Gutierrez C. Influence of Genetic Polymorphisms of Tumor Necrosis Factor Alpha and Interleukin 10 Genes on the Risk of Liver Cirrhosis in HIV-HCV Coinfected Patients [Internet]. PLoS ONE. J.-A. Giron-Gonzalez, Unidad de Enfermedades Infecciosas, Hospital Universitario Puerta del Mar, Cadiz, Spain. E-mail: joseantonio.giron@uca.es: Public Library of Science (185 Berry Street, Suite 1300, San Francisco CA 94107, United States); 2013. p. no pagination. Available from: http://www.plosone.org/article/fetchObject.action?uri=info%3Adoi%2F10.1371%2Fjournal.pone.0066619&representation=PDF

Corradini SG, Burza MA, Molinaro A, Romeo S. Patatin-like phospholipase domain containing 3 sequence variant and hepatocellular carcinoma. Hepatology [Internet]. Wiley Subscription Services, Inc., A Wiley Company; 2011 May [cited 2016 Dec 7];53(5):1776–1776. Available from: http://doi.wiley.com/10.1002/hep.24244

Curto TM, Lagier RJ, Lok AS, Everhart JE, Rowland CM, Sninsky JJ, et al. Predicting cirrhosis and clinical outcomes in patients with advanced chronic hepatitis C with a panel of genetic markers (CRS7). Pharmacogenet Genomics [Internet]. United States: Curto,Teresa M. New England Research Institutes, Inc., Watertown, Massachusetts 02472, USA. tcurto@neriscience.com; 2011 Dec [cited 2016 Apr 25];21(12):851–60. Available from: http://www.pubmedcentral.nih.gov/articlerender.fcgi?artid=3215092&tool=pmcentrez&rendertype=abstract

Dai C-Y, Chuang W-L, Hsieh M-Y, Lee L-P, Hou N-J, Chen S-C, et al. Polymorphism of interferon-gamma gene at position +874 and clinical characteristics of chronic hepatitis C. Transl Res [Internet]. United States: Dai,Chia-Yen. Division of Gastroenterology, Faculty of Medicine, College of Medicine, Kaohsiung Medical University, Kaohsiung, Taiwan.; 2006;148(3):128–33. Available from: http://ovidsp.ovid.com/ovidweb.cgi?T=JS&PAGE=reference&D=med5&NEWS=N&AN=16938650

Dai C-Y, Chuang W-L, Lee L-P, Chen S-C, Hou N-J, Lin Z-Y, et al. Associations of tumour necrosis factor alpha promoter polymorphisms at position -308 and -238 with clinical characteristics of chronic hepatitis C. J Viral Hepat [Internet]. England: Dai,C-Y. Division of Hepatobiliary, Faculty of Medicine, College of Medicine, Kaohsiung Medical University Hospital, No. 100 Tzyou 1st Road, Kaohsiung 807, Taiwan.; 2006;13(11):770–4. Available from: http://ovidsp.ovid.com/ovidweb.cgi?T=JS&PAGE=reference&D=med5&NEWS=N&AN=17052277

De Re V., Caggiari L., De Zorzi M., Repetto O., Zignego A.L., Izzo F., et al. Genetic diversity of the KIR/HLA system and susceptibility to hepatitis C virus-related diseases [Internet]. PLoS ONE. United States: Public Library of Science; 2015. p. no pagination. Available from: http://www.plosone.org/article/fetchObject.action?uri=info:doi/10.1371/journal.pone.0117420&representation=PDF

De Re V, Gragnani L, Fognani E, Piluso A, Izzo F, Mangia A, et al. Impact of immunogenetic IL28B polymorphism on natural outcome of HCV infection. Biomed Res Int [Internet]. United States: Hindawi Publishing Corporation (410 Park Avenue, 15th Floor, 287 pmb, New York NY 10022, United States); 2014;2014:no pagination. Available from: http://www.hindawi.com/journals/biomed/

Deghady A, Abdou A, El-Neanaey WA, Diab I. Association of Genetic Polymorphism -670A>G in the Fas Gene and Serum Markers AST Platelet Ratio Index, AST/ALT with Significant Fibrosis and Cirrhosis in Chronic Hepatitis C. Genet Test Mol Biomarkers [Internet]. United States: Deghady,Akram. Department of Clinical Pathology, Faculty of Medicine, Alexandria University, Alexandria, Egypt.; 2012;16(6):531–5. Available from: http://www.ncbi.nlm.nih.gov/entrez/query.fcgi?cmd=Retrieve&db=PubMed&dopt=Citation&list_uids=22352690

El Bassuoni MA, Soliman MA, El Megeed NA, Al Gazar A. IL-17 Producing Cells and RORγt mRNA Transcriptional Factor in Cirrhotic and HCC Egyptian Patients. Egypt J Immunol [Internet]. 2015 [cited 2016 Dec 7];22(1):59–68. Available from: http://www.ncbi.nlm.nih.gov/pubmed/26415373

El Samanoudy A, Monir R, Badawy A, Ibrahim L, Farag K, El Baz S, et al. Matrix metalloproteinase-9 gene polymorphism in hepatocellular carcinoma patients with hepatitis B and C viruses. Genet Mol Res [Internet]. Brazil: El Samanoudy,A. Department of Medical Biochemistry, Mansoura University, Faculty of Medicine, Mansoura, Egypt.; 2014;13(3):8025–34. Available from: http://ovidsp.ovid.com/ovidweb.cgi?T=JS&PAGE=reference&D=medl&NEWS=N&AN=25299117

El-Awady MK, Mostafa L, Tabll AA, Abdelhafez TH, El Din NGB, Zayed N, et al. Association of il28b snp with progression of egyptian hcv genotype 4 patients to end stage liver disease [Internet]. Hepatitis Monthly. M. K. El-awady, Department of Microbial Biotechnology (Biomedical Technology group) National Research Center, El-Behooth Street 12622m Dokki, Giza, Egypt. E-mail: mkawady@yahoo.com: Kowsar Publishing Company; 2012. p. 271–7. Available from: http://hepatmon.com/?page=download&file_id=8004

ELBassuoni M.A.E.R., Abd El Fatah G. IL17A gene polymorphism, serum IL17 and total IgE in Egyptian population with chronic HCV and hepatocellular carcinoma [Internet]. Immunology Letters. G. Abd El Fatah, Clinical Pathology Department, Menoufia Univerisity, Shebin Elkom, Egypt. E-mail: dr.gehansh7977@gmail.com: Elsevier; 2015. p. 240–5. Available from: http://www.elsevier.com/locate/immlet

Eurich D, Boas-Knoop S, Morawietz L, Neuhaus R, Somasundaram R, Ruehl M, et al. Association of mannose-binding lectin-2 gene polymorphism with the development of hepatitis C-induced hepatocellular carcinoma. Liver Int [Internet]. England: Eurich,Dennis. General, Visceral and Transplantation Surgery, Charite Campus Virchow, Berlin, Germany. dennis.eurich@charite.de; 2011;31(7):1006–12. Available from: http://ovidsp.ovid.com/ovidweb.cgi?T=JS&PAGE=reference&D=med5&NEWS=N&AN=21733090

Ezzikouri S, Alaoui R, Rebbani K, Brahim I, Fakhir F-Z, Nadir S, et al. Genetic variation in the interleukin-28B gene is associated with spontaneous clearance and progression of hepatitis C virus in Moroccan patients. PLoS One [Internet]. United States: Ezzikouri,Sayeh. Virology Unit, Viral Hepatitis Laboratory, Pasteur Institute of Morocco, Casablanca, Morocco. sayeh.ezzikouri@pasteur.ma; 2013;8(1):e54793. Available from: http://ovidsp.ovid.com/ovidweb.cgi?T=JS&PAGE=reference&D=medl&NEWS=N&AN=23358556

Ezzikouri S, Alaoui R, Tazi S, Nadir S, Elmdaghri N, Pineau P, et al. The adiponutrin I148M variant is a risk factor for HCV-associated liver cancer in North-African patients. Infect Genet Evol [Internet]. Netherlands: Ezzikouri,Sayeh. Virology Unit, Viral Hepatitis Laboratory, Pasteur Institute of Morocco, Casablanca, Morocco. Electronic address: sayeh.ezzikouri@pasteur.ma.; 2014;21:179–83. Available from: http://ovidsp.ovid.com/ovidweb.cgi?T=JS&PAGE=reference&D=medl&NEWS=N&AN=24269995

Ezzikouri S, El Feydi AE, Afifi R, Benazzouz M, Hassar M, Pineau P, et al. Polymorphisms in antioxidant defence genes and susceptibility to hepatocellular carcinoma in a Moroccan population. Free Radic Res [Internet]. England: Ezzikouri,Sayeh. Laboratoire des Hepatites Virales, Institut Pasteur du Maroc, 20360 Casablanca, Morocco. sayeh.ezzikouri@pasteur.ma; 2010;44(2):208–16. Available from: http://ovidsp.ovid.com/ovidweb.cgi?T=JS&PAGE=reference&D=med5&NEWS=N&AN=19929244

Ezzikouri S, El Feydi AE, Afifi R, El Kihal L, Benazzouz M, Hassar M, et al. MDM2 SNP309T>G polymorphism and risk of hepatocellular carcinoma: a case-control analysis in a Moroccan population. Cancer Detect Prev [Internet]. England: Ezzikouri,Sayeh. Laboratoire de Virologie, Institut Pasteur du Maroc, Casablanca, Morocco. sayeh.ezzikouri@pasteur.ma; 2009;32(5-6):380–5. Available from: http://ovidsp.ovid.com/ovidweb.cgi?T=JS&PAGE=reference&D=med5&NEWS=N&AN=19233569

Ezzikouri S, El Feydi AE, Benazzouz M, Afifi R, El Kihal L, Hassar M, et al. Single nucleotide polymorphism in DNMT3B promoter and its association with hepatocellular carcinoma in a Moroccan population. Infect Genet Evol [Internet]. Netherlands: Ezzikouri,Sayeh. Laboratoire des Hepatites Virales, Institut Pasteur du Maroc 1, Place Louis Pasteur, 20100 Casablanca, Morocco. sayeh.ezzikouri@pasteur.ma; 2009;9(5):877–81. Available from: http://ovidsp.ovid.com/ovidweb.cgi?T=JS&PAGE=reference&D=med5&NEWS=N&AN=19465161

Ezzikouri S, El Feydi AE, Chafik A, Afifi R, El Kihal L, Benazzouz M, et al. Genetic polymorphism in the manganese superoxide dismutase gene is associated with an increased risk for hepatocellular carcinoma in HCV-infected Moroccan patients. Mutat Res [Internet]. Netherlands: Ezzikouri,Sayeh. Laboratoire de Virologie, Institut Pasteur du Maroc 1, Place Louis Pasteur, 20100, Casablanca, Morocco.; 2008;649(1-2):1–6. Available from: http://ovidsp.ovid.com/ovidweb.cgi?T=JS&PAGE=reference&D=med5&NEWS=N&AN=18023606

Ezzikouri S, El feydi AE, Chafik A, Benazzouz M, El kihal L, Afifi R, et al. The Pro variant of the p53 codon 72 polymorphism is associated with hepatocellular carcinoma in Moroccan population [Internet]. Hepatology Research S. Benjelloun, Laboratoire de Virologie, Institut Pasteur du Maroc 1, Place Louis Pasteur, 20100 Casablanca, Morocco. E-mail: soumaya.benjelloun@pasteur.ma: Blackwell Publishing Inc.; Sep, 2007 p. 748–54. Available from: http://doi.wiley.com/10.1111/j.1872-034X.2007.00126.x

Fabris C, Falleti E, Cussigh A, Bitetto D, Fontanini E, Bignulin S, et al. IL-28B rs12979860 C/T allele distribution in patients with liver cirrhosis: role in the course of chronic viral hepatitis and the development of HCC. J Hepatol [Internet]. England: Fabris,Carlo. Internal Medicine, DPMSC, University of Udine, Italy.; 2011 [cited 2016 Dec 7];54(4):716–22. Available from: http://ovidsp.ovid.com/ovidweb.cgi?T=JS&PAGE=reference&D=med5&NEWS=N&AN=21146242

Falleti E, Cmet S, Fabris C, Bitetto D, Cussigh A, Fornasiere E, et al. Association between the epidermal growth factor rs4444903 G/G genotype and advanced fibrosis at a young age in chronic hepatitis C. Cytokine [Internet]. United States: Falleti,Edmondo. Department of Laboratory Medicine, University of Udine, Italy.; 2012;57(1):68–73. Available from: http://ovidsp.ovid.com/ovidweb.cgi?T=JS&PAGE=reference&D=medl&NEWS=N&AN=22122913

Fishman S, Lurie Y, Peretz H, Morad T, Grynberg E, Blendis LM, et al. Role of CYP2D6 polymorphism in predicting liver fibrosis progression rate in Caucasian patients with chronic hepatitis C. Liver Int [Internet]. England: Fishman,Sigal. Liver Unit, Gastroenterology Institute, Tel Aviv Sourasky Medical Center, Affiliated to Sackler School of Medicine, Tel Aviv, Israel. sigal001right@gmail.com; 2006;26(3):279–84. Available from: http://ovidsp.ovid.com/ovidweb.cgi?T=JS&PAGE=reference&D=med5&NEWS=N&AN=16584388

Fontana RJ, Litman HJ, Dienstag JL, Bonkovsky HL, Su G, Sterling RK, et al. YKL-40 genetic polymorphisms and the risk of liver disease progression in patients with advanced fibrosis due to chronic hepatitis C. Liver Int [Internet]. England: Fontana,Robert J. Department of Internal Medicine, University of Michigan Medical School, Ann Arbor, MI 48109-0362, USA. rfontana@med.umich.edu; 2012;32(4):665–74. Available from: http://ovidsp.ovid.com/ovidweb.cgi?T=JS&PAGE=reference&D=medl&NEWS=N&AN=22103814

Gehrke SG, Stremmel W, Mathes I, Riedel H-D, Bents K, Kallinowski B. Hemochromatosis and transferrin receptor gene polymorphisms in chronic hepatitis C: impact on iron status, liver injury and HCV genotype. J Mol Med (Berl) [Internet]. Germany: Gehrke,Sven G. Department of Internal Medicine IV, University Hospital of Heidelberg, Bergheimer Strasse 58, 69115 Heidelberg, Germany.; 2003;81(12):780–7. Available from: http://ovidsp.ovid.com/ovidweb.cgi?T=JS&PAGE=reference&D=med4&NEWS=N&AN=14557859

Geier A, Reugels M, Weiskirchen R, Wasmuth HE, Dietrich CG, Siewert E, et al. Common heterozygous hemochromatosis gene mutations are risk factors for inflammation and fibrosis in chronic hepatitis C. Liver Int [Internet]. England: Geier,Andreas. Department of Medicine III, University Hospital Aachen, Aachen University (RWTH), Germany.; 2004;24(4):285–94. Available from: http://ovidsp.ovid.com/ovidweb.cgi?T=JS&PAGE=reference&D=med5&NEWS=N&AN=15287851

Guo P-F, Jin J, Sun X. Influence of IL10 gene polymorphisms on the severity of liver fibrosis and susceptibility to liver cirrhosis in HBV/HCV-infected patients. Infect Genet Evol [Internet]. Netherlands: Guo,Peng-Fei. Department of Mathematics, South China University of Technology, Guangzhou, China. Electronic address: guo.pf@mail.scut.edu.cn.; 2015;30:89–95. Available from: http://ovidsp.ovid.com/ovidweb.cgi?T=JS&PAGE=reference&D=medl&NEWS=N&AN=25514046

Halangk J, Sarrazin C, Neumann K, Puhl G, Mueller T, Teuber G, et al. Evaluation of complement factor 5 variants as genetic risk factors for the development of advanced fibrosis in chronic hepatitis C infection. J Hepatol [Internet]. England: Halangk,Juliane. Medizinische Klinik mit Schwerpunkt Hepatologie und Gastroenterologie, Charite - Universitatsmedizin Berlin, Campus Virchow-Klinikum, Germany.; 2008;49(3):339–45. Available from: http://ovidsp.ovid.com/ovidweb.cgi?T=JS&PAGE=reference&D=med5&NEWS=N&AN=18644651

Halla MC, do Carmo RF, Silva Vasconcelos LR, Pereira LB, Moura P, de Siqueira ERF, et al. Association of hepatitis C virus infection and liver fibrosis severity with the variants alleles of MBL2 gene in a Brazilian population. Hum Immunol [Internet]. United States: Halla,Maria Cristina. Postgraduate Program RENORBIO, Brazil; Institute of Biological Sciences, University of Pernambuco, Recife, Brazil.; 2010;71(9):883–7. Available from: http://ovidsp.ovid.com/ovidweb.cgi?T=JS&PAGE=reference&D=med5&NEWS=N&AN=20570631

Hamaoka.K., Nagoshi.S., Sugawara.K., Naiki.K., Uchida.Y., Inao.M., et al. SNPs in the promoter region of the osteopontin gene as a possible host factor for sex difference in hepatocellular carcinoma development in patients with HCV [Internet]. Hepatology International. S. Mochida, Department of Gastroenterology and Hepatology, Saitama Medical University, 38 Morohongo, Moroyama-machi, Iruma-gun, Saitama 350-0495, Japan. E-mail: smochida@saitama-med.ac.jp: Springer New York (233 Spring Street, New York NY 10013-1578, United States); 2013. p. 683–92. Available from: http://ovidsp.ovid.com/ovidweb.cgi?T=JS&PAGE=reference&D=emed11&NEWS=N&AN=2013423784

He J, Yu G, Li Z, Liang H. Influence of interleukin-28B polymorphism on progression to hepatitis virus-induced hepatocellular carcinoma. Tumour Biol [Internet]. Netherlands: He,Jinxia. Department of Oncology and Southwest Cancer Center, Southwest Hospital, Third Military Medical University, Chongqing, 400038, China.; 2014;35(9):8757–63. Available from: http://ovidsp.ovid.com/ovidweb.cgi?T=JS&PAGE=reference&D=medl&NEWS=N&AN=24874053

Hsieh Y-S, Tsai C-M, Yeh C-B, Yang S-F, Hsieh Y-H, Weng C-J. Survivin T9809C, an SNP located in 3’-UTR, displays a correlation with the risk and clinicopathological development of hepatocellular carcinoma. Ann Surg Oncol [Internet]. United States: Hsieh,Yih-Shou. Institute of Biochemistry and Biotechnology, Chung Shan Medical University, Taichung, Taiwan.; 2012;19 Suppl 3:S625–33. Available from: http://ovidsp.ovid.com/ovidweb.cgi?T=JS&PAGE=reference&D=medl&NEWS=N&AN=22052111

Huang H, Shiffman ML, Cheung RC, Layden TJ, Friedman S, Abar OT, et al. Identification of two gene variants associated with risk of advanced fibrosis in patients with chronic hepatitis C. Gastroenterology [Internet]. United States: Huang,Hongjin. Celera Diagnostics, Alameda, California, USA. hongjin.huang@celera.diagnostics.com; 2006;130(6):1679–87. Available from: http://ovidsp.ovid.com/ovidweb.cgi?T=JS&PAGE=reference&D=med5&NEWS=N&AN=16697732

Hue S, Cacoub P, Renou C, Halfon P, Thibault V, Charlotte F, et al. Human leukocyte antigen class II alleles may contribute to the severity of hepatitis C virus-related liver disease. J Infect Dis [Internet]. United States: Hue,Sophie. Laboratory of Immunology, Hopital Necker, Paris, France.; 2002;186(1):106–9. Available from: http://ovidsp.ovid.com/ovidweb.cgi?T=JS&PAGE=reference&D=med4&NEWS=N&AN=12089669

Hung C.-H., Chiu Y.-C., Hu T.-H., Chen C.-H., Lu S.-N., Huang C.-M., et al. Significance of vitamin D receptor gene polymorphisms for risk of hepatocellular carcinoma in chronic hepatitis C [Internet]. Translational Oncology. C.-H. Hung, Division of Hepatogastroenterology, Department of Internal Medicine, Kaohsiung Chang Gung Memorial Hospital, 123 Ta Pei Road, Niao Sung 833 Kaohsiung, Taiwan (Republic of China). E-mail: chh4366@yahoo.com.tw: Translational Oncology Editorial Office; 2014. p. 503–7. Available from: http://www.transonc.com/article/S1936-5233(14)00056-4/pdf

Ibrahim A, El-Azim SA. Association of MnSOD Ala16Val genotype and activity with hepatocellular carcinoma risk in HCV-infected Egyptian patients [Internet]. Arab Journal of Gastroenterology. S.A. El-Azim, Microbiology and Immunology Department, Faculty of Medicine, Zagazig University, Zagazig, Egypt. E-mail: shymaa_abdelazim@yahoo.com: Elsevier Ltd (Langford Lane, Kidlington, Oxford OX5 1GB, United Kingdom); 2010. p. 19–23. Available from: http://ovidsp.ovid.com/ovidweb.cgi?T=JS&PAGE=reference&D=emed9&NEWS=N&AN=2010423151

Ikebuchi Y, Ishida C, Okamoto K, Murawaki Y. Association of TIMP-1 and TIMP-2 gene polymorphisms with progression of liver fibrosis in patients with type C chronic liver disease. Biochem Genet [Internet]. United States: Ikebuchi,Yuichiro. Division of Medicine and Clinical Science, Department of Multidisciplinary Internal Medicine, Tottori University School of Medicine, Nishi-cho 36-1, Yonago 683-8504, Japan. ikebu@med.tottori-u.ac.jp; 2013;51(7-8):564–74. Available from: http://ovidsp.ovid.com/ovidweb.cgi?T=JS&PAGE=reference&D=medl&NEWS=N&AN=23563628

Ishida C, Ikebuchi Y, Okamoto K, Murawaki Y. Functional gene polymorphisms of interleukin-10 are associated with liver disease progression in Japanese patients with hepatitis C virus infection. Intern Med [Internet]. Japan: Ishida,Chihiro. Department of Multidisciplinary Internal Medicine, Tottori University School of Medicine, Japan. lanze@med.tottori-u.ac.jp; 2011;50(7):659–66. Available from: http://ovidsp.ovid.com/ovidweb.cgi?T=JS&PAGE=reference&D=med5&NEWS=N&AN=21467695

Iwata R, Baur K, Stieger B, Mertens JC, Daly AK, Frei P, et al. A common polymorphism in the ABCB11 gene is associated with advanced fibrosis in hepatitis C but not in non-alcoholic fatty liver disease. Clin Sci (Lond) [Internet]. England: Iwata,Rika. Department of Internal Medicine, Division of Gastroenterology and Hepatology, University Hospital Zurich (USZ), CH-8091 Zurich, Switzerland.; 2011;120(7):287–96. Available from: http://ovidsp.ovid.com/ovidweb.cgi?T=JS&PAGE=reference&D=med5&NEWS=N&AN=20883210

Jeng J-E, Tsai J-F, Chuang L-Y, Ho M-S, Lin Z-Y, Hsieh M-Y, et al. Heat shock protein A1B 1267 polymorphism is highly associated with risk and prognosis of hepatocellular carcinoma: a case-control study. Medicine (Baltimore) [Internet]. United States: Jeng,Jen-Eing. Department of Clinical Laboratory, Faculty of Medicine, College of Medicine, Kaohsiung Medical University, Kaohsiung, Taiwan.; 2008;87(2):87–98. Available from: http://ovidsp.ovid.com/ovidweb.cgi?T=JS&PAGE=reference&D=med5&NEWS=N&AN=18344806

Jeng J-E, Tsai J-F, Chuang L-Y, Ho M-S, Lin Z-Y, Hsieh M-Y, et al. Tumor necrosis factor-alpha 308.2 polymorphism is associated with advanced hepatic fibrosis and higher risk for hepatocellular carcinoma. Neoplasia [Internet]. Canada: Jeng,Jen-Eing. Department of Clinical Laboratory, Kaohsiung Medical University Hospital, Kaohsiung, Taiwan.; 2007;9(11):987–92. Available from: http://ovidsp.ovid.com/ovidweb.cgi?T=JS&PAGE=reference&D=med5&NEWS=N&AN=18030367

Jeng J-E, Wu H-F, Tsai M-F, Tsai H-R, Chuang L-Y, Lin Z-Y, et al. Independent and additive interaction between tumor necrosis factor beta +252 polymorphisms and chronic hepatitis B and C virus infection on risk and prognosis of hepatocellular carcinoma: a case-control study. Asian Pac J Cancer Prev [Internet]. Thailand: Jeng,Jen-Eing. Department of Laboratory Medicine, Kaohsiung Medical University Hospital, Kaohsiung Medical University, Kaohsiung, Taiwan E-mail : jftsai@cc.kmu.edu.tw.; 2014;15(23):10209–15. Available from: http://ovidsp.ovid.com/ovidweb.cgi?T=JS&PAGE=reference&D=medl&NEWS=N&AN=25556449

Jia S, Tang W. P53 codon 72 polymorphism and hepatocellular carcinoma: A meta-analysis [Internet]. Hepatology International. S. Jia, Laboratory of Molecular Genetics of Aging and Tumor, Faculty of Environmental Science and Engineering, Kunming University of Science and Technology, 727 Jing Ming Nan Road, Kunming 650500 Yunnan, China. E-mail: lilith-jia@hotmail.com: Springer New York (233 Spring Street, New York NY 10013-1578, United States); 2013. p. 669–75. Available from: http://ovidsp.ovid.com/ovidweb.cgi?T=JS&PAGE=reference&D=emed11&NEWS=N&AN=2013423774

Jia Z-F, Su H-Y, Li X-L, Xu X, Yin Z-H, Guan P. Polymorphisms of UGT1A7 and XRCC1 are associated with an increased risk of hepatocellular carcinoma in Northeast China. Chinese J Cancer Res [Internet]. B.-S. Zhou, Department of Epidemiology, School of Public Health, China Medical University, Shenyang 110001, China. E-mail: bszhou@mail.cmu.edu.cn: Beijing Institute for Cancer Research (Da-Hong-Luo-Chang Street, Western District, Beijing 100034, China); 2010 Dec 26 [cited 2016 Nov 3];22(4):260–6. Available from: http://link.springer.com/10.1007/s11670-010-0260-z

Joshita S, Umemura T, Katsuyama Y, Ichikawa Y, Kimura T, Morita S, et al. Association of IL28B gene polymorphism with development of hepatocellular carcinoma in Japanese patients with chronic hepatitis C virus infection. Hum Immunol [Internet]. United States: Joshita,Satoru. Department of Medicine, Division of Gastroenterology and Hepatology, Shinshu University School of Medicine, Matsumoto, Japan.; 2012;73(3):298–300. Available from: http://ovidsp.ovid.com/ovidweb.cgi?T=JS&PAGE=reference&D=medl&NEWS=N&AN=22245236

Kato N, Ji G, Wang Y, Baba M, Hoshida Y, Otsuka M, et al. Large-scale search of single nucleotide polymorphisms for hepatocellular carcinoma susceptibility genes in patients with hepatitis C. Hepatology [Internet]. United States: Kato,Naoya. Department of Gastroenterology, Graduate School of Medicine, University of Tokyo, Tokyo, Japan. kato-2im@h.u-tokyo.ac.jp; 2005;42(4):846–53. Available from: http://ovidsp.ovid.com/ovidweb.cgi?T=JS&PAGE=reference&D=med5&NEWS=N&AN=16175604

Kato S, Tajiri T, Matsukura N, Matsuda N, Taniai N, Mamada H, et al. Genetic polymorphisms of aldehyde dehydrogenase 2, cytochrome p450 2E1 for liver cancer risk in HCV antibody-positive japanese patients and the variations of CYP2E1 mRNA expression levels in the liver due to its polymorphism. Scand J Gastroenterol [Internet]. Norway: Kato,S. Surgery for Organ Function and Biological Regulation, Nippon Medical School, Tokyo, Japan. katoshun@nms.ac.jp; 2003;38(8):886–93. Available from: http://ovidsp.ovid.com/ovidweb.cgi?T=JS&PAGE=reference&D=med4&NEWS=N&AN=12940444

Korner C, Riesner K, Kramer B, Eisenhardt M, Glassner A, Wolter F, et al. TRAIL receptor I (DR4) polymorphisms C626G and A683C are associated with an increased risk for hepatocellular carcinoma (HCC) in HCV-infected patients. BMC Cancer [Internet]. England: Korner,Christian. Department of Internal Medicine I, University of Bonn, Sigmund-Freud-Str, 25, 53127 Bonn, Germany.; 2012;12:no pagination. Available from: http://www.biomedcentral.com/1471-2407/12/85

Kumar V, Kato N, Urabe Y, Takahashi A, Muroyama R, Hosono N, et al. Genome-wide association study identifies a susceptibility locus for HCV-induced hepatocellular carcinoma. Nat Genet [Internet]. United States: Kumar,Vinod. Laboratory of Molecular Medicine, Human Genome Center, Institute of Medical Science, University of Tokyo, Tokyo, Japan.; 2011;43(5):455–8. Available from: http://ovidsp.ovid.com/ovidweb.cgi?T=JS&PAGE=reference&D=med5&NEWS=N&AN=21499248

Labib HA, Ahmed HS, Shalaby SM, Wahab EA, Hamed EF. Genetic polymorphism of IL-23R influences susceptibility to HCV-related hepatocellular carcinoma. Cell Immunol [Internet]. United States: Labib,Hany A. Clinical Pathology Department, Faculty of Medicine, Zagazig University, Zagazig, Egypt.; 2015;294(1):21–4. Available from: http://ovidsp.ovid.com/ovidweb.cgi?T=JS&PAGE=reference&D=medl&NEWS=N&AN=25666505

Lange CM, Bibert S, Dufour J-F, Cellerai C, Cerny A, Heim MH, et al. Comparative genetic analyses point to HCP5 as susceptibility locus for HCV-associated hepatocellular carcinoma. J Hepatol [Internet]. Netherlands: Lange,Christian M. Division of Gastroenterology and Hepatology, University Hospital Lausanne, CH-1011 Lausanne, Switzerland. Christian.Lange@kgu.de; 2013;59(3):504–9. Available from: http://ovidsp.ovid.com/ovidweb.cgi?T=JS&PAGE=reference&D=medl&NEWS=N&AN=23665287

Lange CM, Miki D, Ochi H, Nischalke H-D, Bojunga J, Bibert S, et al. Genetic analyses reveal a role for vitamin D insufficiency in HCV-associated hepatocellular carcinoma development. PLoS One [Internet]. United States: Lange,Christian M. Division of Gastroenterology and Hepatology, Centre Hospitalier Universitaire Vaudois, University of Lausanne, Lausanne, Switzerland. lange_christian1@yahoo.de; 2013;8(5):e64053. Available from: http://ovidsp.ovid.com/ovidweb.cgi?T=JS&PAGE=reference&D=medl&NEWS=N&AN=23734184

Lauret E, Rodriguez M, Gonzalez S, Linares A, Lopez-Vazquez A, Martinez-Borra J, et al. HFE gene mutations in alcoholic and virus-related cirrhotic patients with hepatocellular carcinoma. Am J Gastroenterol [Internet]. United States: Lauret,Eugenia. Department of Gastroenterology, Hospital Central de Asturias, Spain.; 2002;97(4):1016–21. Available from: http://ovidsp.ovid.com/ovidweb.cgi?T=JS&PAGE=reference&D=med4&NEWS=N&AN=12003382

Leveri M, Gritti C, Rossi L, Zavaglia C, Civardi E, Mondelli MU, et al. Codon 72 polymorphism of P53 gene does not affect the risk of cirrhosis and hepatocarcinoma in HCV-infected patients. Cancer Lett [Internet]. Ireland: Leveri,Michela. ASAEV-Associazione Studio Avanzato Epatiti Virali, via Garibaldi 13, 24040 Bonate Sotto. BG, Italy. michela.asaev@tiscali.it; 2004;208(1):75–9. Available from: http://ovidsp.ovid.com/ovidweb.cgi?T=JS&PAGE=reference&D=med5&NEWS=N&AN=15105048

Li C-Z, Kato N, Chang J-H, Muroyama R, Shao R-X, Dharel N, et al. Polymorphism of OAS-1 determines liver fibrosis progression in hepatitis C by reduced ability to inhibit viral replication. Liver Int [Internet]. England: Li,Chang-Zheng. Department of Gastroenterology, Graduate School of Medicine, University of Tokyo,4-6-1 Shirokanedai, Minato-ku, Tokyo, Japan.; 2009;29(9):1413–21. Available from: http://ovidsp.ovid.com/ovidweb.cgi?T=JS&PAGE=reference&D=med5&NEWS=N&AN=19515215

Li Y, Wang K., Dai L., Wang P., Song C., Shi J., et al. HapMap-based study of CIP2A gene polymorphisms and HCC susceptibility [Internet]. Oncology Letters. J. Zhang, Department of Epidemiology, College of Public Health, Zhengzhou University, 100 Science Ave, Zhengzhou 450001, China. E-mail: jianyingzhang@hotmail.com: Spandidos Publications Ltd. (10 Vriaxidos Street, Athens 11635, Greece); 2012. p. 358–64. Available from: http://www.spandidos-publications.com/serveFile/ol.2012.728_AOP_PDF.pdf?type=article&article_id=ol_4_2_358&item=PDF

Littera R, Zamboni F, Tondolo V, Fantola G, Chessa L, Orru N, et al. Absence of activating killer immunoglobulin-like receptor genes combined with hepatitis C viral genotype is predictive of hepatocellular carcinoma. Hum Immunol [Internet]. United States: Littera,Roberto. Centro Regionale Trapianti, Ospedale R. Binaghi - ASL 8, 09126 Cagliari, Italy. Electronic address: roby.litter@gmail.com.; 2013;74(10):1288–94. Available from: http://ovidsp.ovid.com/ovidweb.cgi?T=JS&PAGE=reference&D=medl&NEWS=N&AN=23756163

Liu Y, El-Serag HB, Jiao L, Lee J, Moore D, Franco LM, et al. WNT signaling pathway gene polymorphisms and risk of hepatic fibrosis and inflammation in HCV-infected patients. PLoS One [Internet]. Department of Pediatrics, Baylor College of Medicine, Houston, TX, United States: Liu,Yanhong. Department of Pediatrics, Baylor College of Medicine, Houston, Texas, United States of America ; Dan L. Duncan Cancer Center at Baylor College of Medicine, Houston, Texas, United States of America.; 2013;8(12):no pagination. Available from: http://www.plosone.org/article/fetchObject.action?uri=info%3Adoi%2F10.1371%2Fjournal.pone.0084407&representation=PDF

Lo PHY, Urabe Y, Kumar V, Tanikawa C, Koike K, Kato N, et al. Identification of a functional variant in the MICA promoter which regulates MICA expression and increases HCV-related hepatocellular carcinoma risk. PLoS One [Internet]. United States: Public Library of Science (185 Berry Street, Suite 1300, San Francisco CA 94107, United States); 2013;8(4):e61279. Available from: http://ovidsp.ovid.com/ovidweb.cgi?T=JS&PAGE=reference&D=medl&NEWS=N&AN=23593449

Lopez-Vazquez A, Rodrigo L, Martinez-Borra J, Perez R, Rodriguez M, Fdez-Morera JL, et al. Protective effect of the HLA-Bw4I80 epitope and the killer cell immunoglobulin-like receptor 3DS1 gene against the development of hepatocellular carcinoma in patients with hepatitis C virus infection. J Infect Dis [Internet]. United States: Lopez-Vazquez,Antonio. Department of Immunology, Hospital Universitario Central de Asturias, Oviedo, Spain.; 2005;192(1):162–5. Available from: http://ovidsp.ovid.com/ovidweb.cgi?T=JS&PAGE=reference&D=med5&NEWS=N&AN=15942906

Lopez-Vazquez A, Rodrigo L, Mina-Blanco A, Martinez-Borra J, Fuentes D, Rodriguez M, et al. Extended human leukocyte antigen haplotype EH18.1 influences progression to hepatocellular carcinoma in patients with hepatitis C virus infection. J Infect Dis [Internet]. United States: Lopez-Vazquez,Antonio. Department of Immunology, Hospital Central de Asturias, Oviedo, Spain.; 2004;189(6):957–63. Available from: http://ovidsp.ovid.com/ovidweb.cgi?T=JS&PAGE=reference&D=med5&NEWS=N&AN=14999597

Lundbo LF, Clausen LN, Weis N, Schonning K, Rosenorn L, Benfield T, et al. Influence of hepatitis C virus and IL28B genotypes on liver stiffness. PLoS One [Internet]. United States: Lundbo,Lene Fogt. Department of Infectious Diseases, Hvidovre Hospital, University of Copenhagen, Copenhagen, Denmark; Clinical Research Centre, Hvidovre Hospital, University of Copenhagen, Copenhagen, Denmark; Department of Clinical Medicine, Faculty of ; 2014;9(12):e115882. Available from: http://ovidsp.ovid.com/ovidweb.cgi?T=JS&PAGE=reference&D=medl&NEWS=N&AN=25545640

Ma J, Liu YC, Fang Y, Cao Y. TGF-beta1 polymorphism 509 C>T is associated with an increased risk for hepatocellular carcinoma in HCV-infected patients [Internet]. Genetics and Molecular Research. Z.L. Liu, Department of Clinical Laboratory, The First People’s Hospital of Lianyungang, Lianyungang, China: Fundacao de Pesquisas Cientificas de Ribeirao Preto; 2015. p. 4461–8. Available from: http://www.geneticsmr.com//year2015/vol14-2/pdf/gmr4118.pdf

Mah Y.-H., Hsu C.-S., Liu C.-H., Liu C.-J., Lai M.-Y., Chen P.-J., et al. Serum p53 gene polymorphisms and severity of hepatitis B or C-related chronic liver diseases in Taiwan [Internet]. Hepatology International. J.-H. Kao, Graduate Institute of Clinical Medicine, National Taiwan University, College of Medicine, 1 Chang-Te St., Taipei 100, Taiwan (Republic of China). E-mail: kaojh@ntu.edu.tw: Springer New York (233 Springer Street, New York NY 10013-1578, United States); 2011. p. 814–21. Available from: http://ovidsp.ovid.com/ovidweb.cgi?T=JS&PAGE=reference&D=emed10&NEWS=N&AN=2011593095

Maharshak N, Halfon P, Deutsch V, Peretz H, Berliner S, Fishman S, et al. Increased fibrosis progression rates in hepatitis C patients carrying the prothrombin G20210A mutation. World J Gastroenterol [Internet]. China: Maharshak,Nitsan. Department of Gastroenterology and Liver diseases, Tel Aviv Sourasky Medical Center, affiliated to the Sackler School of Medicine, Tel Aviv University, 64239 Tel Aviv, Israel. nitsan_maharshak@walla.com; 2011;17(45):5007–13. Available from: http://ovidsp.ovid.com/ovidweb.cgi?T=JS&PAGE=reference&D=med5&NEWS=N&AN=22174551

Mandai M, Murawaki Y, Okamoto K, Ohtani H, Maeda,N. Association of functional gene polymorphisms of interleukin-1beta and transforming growth factor-beta1 with the progression of liver fibrosis in Japanese patients with hepatitis C virus-related chronic liver disease [Internet]. Yonago Acta Medica. Y. Murawaki, Department of Multidisciplinary Internal Medicine, School of Medicine, Tottori University Faculty of Medicine, Yonago 683-8504, Japan: Tottori University Faculty of Medicine; 2007. p. 89–97. Available from: http://lib.med.tottori-u.ac.jp/yam/yam50-4/50_089-097.pdf

Marcolongo M, Young B, Dal Pero F, Fattovich G, Peraro L, Guido M, et al. A seven-gene signature (cirrhosis risk score) predicts liver fibrosis progression in patients with initially mild chronic hepatitis C. Hepatology [Internet]. United States: Marcolongo,Moira. Department of Histology, Microbiology, and Medical Biotechnologies, University of Padova, Padova, Italy.; 2009;50(4):1038–44. Available from: http://ovidsp.ovid.com/ovidweb.cgi?T=JS&PAGE=reference&D=med5&NEWS=N&AN=19676127

McIlroy D, Theodorou I, Ratziu V, Vidaud D, Pellet P, Debre P, et al. FAS promoter polymorphisms correlate with activity grade in hepatitis C patients. Eur J Gastroenterol Hepatol [Internet]. England: McIlroy,Dorian. Laboratoire d’Immunologie Cellulaire et Tissulaire bService de Gastroenterologie, Hopital Pitie-Salpetriere, Paris, France.; 2005;17(10):1081–8. Available from: http://ovidsp.ovid.com/ovidweb.cgi?T=JS&PAGE=reference&D=med5&NEWS=N&AN=16148554

Medhi S, Sarma MP, Asim M, Kar P. Genetic variants of heat shock protein A1L2437 and A1B1267 as possible risk factors for hepatocellular carcinoma in India. J Viral Hepat [Internet]. England: Medhi,S. Department of Medicine, Maulana Azad Medical College, University of Delhi, New Delhi, India.; 2013;20(4):e141–7. Available from: http://ovidsp.ovid.com/ovidweb.cgi?T=JS&PAGE=reference&D=medl&NEWS=N&AN=23490384

Meiler C, Muhlbauer M, Johann M, Hartmann A, Schnabl B, Wodarz N, et al. Different effects of a CD14 gene polymorphism on disease outcome in patients with alcoholic liver disease and chronic hepatitis C infection. World J Gastroenterol [Internet]. China: Meiler,C. Department of Internal Medicine I, University of Regensburg, Regensburg D-93042, Germany.; 2005;11(38):6031–7. Available from: http://ovidsp.ovid.com/ovidweb.cgi?T=JS&PAGE=reference&D=med5&NEWS=N&AN=16273620

Miki D, Ochi H, Hayes CN, Abe H, Yoshima T, Aikata H, et al. Variation in the DEPDC5 locus is associated with progression to hepatocellular carcinoma in chronic hepatitis C virus carriers. Nat Genet [Internet]. United States: Miki,Daiki. Laboratory for Digestive Diseases, Center for Genomic Medicine, RIKEN, Hiroshima, Japan.; 2011;43(8):797–800. Available from: http://ovidsp.ovid.com/ovidweb.cgi?T=JS&PAGE=reference&D=med5&NEWS=N&AN=21725309

Milano M, Aghemo A, Mancina RM, Fischer J, Dongiovanni P, De Nicola S, et al. Transmembrane 6 superfamily member 2 gene E167K variant impacts on steatosis and liver damage in chronic hepatitis C patients. Hepatology [Internet]. United States: Milano,Marta. Internal Medicine and Metabolic Diseases, Fondazione IRCCS Ca’ Granda Ospedale Maggiore Policlinico, Milan, Italy.; 2015;62(1):111–7. Available from: http://ovidsp.ovid.com/ovidweb.cgi?T=JS&PAGE=reference&D=medl&NEWS=N&AN=25820484

Miyashita M, Ito T, Sakaki M, Kajiwara A, Nozawa H, Hiroishi K, et al. Genetic polymorphism in cyclooxygenase-2 promoter affects hepatic inflammation and fibrosis in patients with chronic hepatitis C. J Viral Hepat [Internet]. England: Miyashita,Miyuki. Division of Gastroenterology, Department of Medicine, Showa University School of Medicine, Tokyo, Japan.; 2012;19(9):608–14. Available from: http://ovidsp.ovid.com/ovidweb.cgi?T=JS&PAGE=reference&D=medl&NEWS=N&AN=22863264

Miyoshi K, Ikebuchi Y, Ishida C, Okamoto K, Murawaki Y. Association between gene polymorphisms of connective tissue growth factor and the progression of chronic liver disease associated with hepatitis C. Intern Med [Internet]. Japan: Miyoshi,Kenichi. Division of Medicine and Clinical Science, Department of Multidisciplinary Internal Medicine, Tottori University School of Medicine, Japan.; 2014;53(14):1461–8. Available from: http://ovidsp.ovid.com/ovidweb.cgi?T=JS&PAGE=reference&D=medl&NEWS=N&AN=25030555

Mochizuki J, Murakami S, Sanjo A, Takagi I, Akizuki S, Ohnishi A. Genetic polymorphisms of cytochrome P450 in patients with hepatitis C virus-associated hepatocellular carcinoma. J Gastroenterol Hepatol [Internet]. Australia: Mochizuki,Junko. Division of Gastroenterology and Hepatology, Department of Internal Medicine, Daisan Hospital, Jikei University School of Medicine, Komae, Tokyo 201-8601, Japan.; 2005;20(8):1191–7. Available from: http://ovidsp.ovid.com/ovidweb.cgi?T=JS&PAGE=reference&D=med5&NEWS=N&AN=16048566

Mohy A, Fouad A. Role of transforming growth factor-β1 in serum and - 509 C>T promoter gene polymorphism in development of liver cirrhosis in Egyptian patients. Meta gene [Internet]. Elsevier; 2014 Dec [cited 2016 Nov 3];2:631–7. Available from: http://www.ncbi.nlm.nih.gov/pubmed/25606446

Motawi TK, Shaker OG, Ismail MF, Sayed NH. Genetic variants associated with the progression of hepatocellular carcinoma in hepatitis C Egyptian patients. Gene [Internet]. Netherlands: Motawi,Tarek Kamal. Biochemistry Department, Faculty of Pharmacy, Cairo University, Cairo, Egypt.; 2013;527(2):516–20. Available from: http://ovidsp.ovid.com/ovidweb.cgi?T=JS&PAGE=reference&D=medl&NEWS=N&AN=23845776

Nahon P, Sutton A, Rufat P, Charnaux N, Mansouri A, Moreau R, et al. A variant in myeloperoxidase promoter hastens the emergence of hepatocellular carcinoma in patients with HCV-related cirrhosis. J Hepatol [Internet]. England: Nahon,Pierre. Service d’Hepatologie, Hopital Jean Verdier, AP-HP, Bondy, France. pierre.nahon@jvr.aphp.fr; 2012;56(2):426–32. Available from: http://ovidsp.ovid.com/ovidweb.cgi?T=JS&PAGE=reference&D=medl&NEWS=N&AN=21907168

Nahon P, Sutton A, Rufat P, Simon C, Trinchet J-C, Gattegno L, et al. Chemokine system polymorphisms, survival and hepatocellular carcinoma occurrence in patients with hepatitis C virus-related cirrhosis. World J Gastroenterol [Internet]. China: Nahon,Pierre. Department of Hepatology, Jean Verdier Hospital AP-HP, Service d’Hepatologie Hospital Jean Verdier Avenue du 14 Juillet, Bondy 93140, France. pierre.nahon@jvr.aphp.fr; 2008;14(5):713–9. Available from: http://ovidsp.ovid.com/ovidweb.cgi?T=JS&PAGE=reference&D=med5&NEWS=N&AN=18205260

Nakamura M, Kanda T, Nakamoto S, Miyamura T, Jiang X, Wu S, et al. No correlation between PNPLA3 rs738409 genotype and fatty liver and hepatic cirrhosis in Japanese patients with HCV. PLoS One [Internet]. Department of Gastroenterology and Nephrology, Chiba University, Graduate School of Medicine, Chiba, Japan: Public Library of Science (185 Berry Street, Suite 1300, San Francisco CA 94107, United States); 2013;8(12):e81312. Available from: http://ovidsp.ovid.com/ovidweb.cgi?T=JS&PAGE=reference&D=medl&NEWS=N&AN=24349054

Nalpas B, Lavialle-Meziani R, Plancoulaine S, Jouanguy E, Nalpas A, Munteanu M, et al. Interferon gamma receptor 2 gene variants are associated with liver fibrosis in patients with chronic hepatitis C infection. Gut [Internet]. England: Nalpas,Bertrand. Unite d’Hepatologie, Institut National de la Sante et de la Recherche Medicale, U567, Hopital Cochin, Paris, France.; 2010;59(8):1120–6. Available from: http://ovidsp.ovid.com/ovidweb.cgi?T=JS&PAGE=reference&D=med5&NEWS=N&AN=20587546

Nischalke HD, Berger C, Luda C, Muller T, Berg T, Coenen M, et al. The CXCL1 rs4074 A allele is associated with enhanced CXCL1 responses to TLR2 ligands and predisposes to cirrhosis in HCV genotype 1-infected Caucasian patients. J Hepatol [Internet]. England: Nischalke,Hans Dieter. Department of Internal Medicine I, University of Bonn, Sigmund-Freud-Str. 25, 53127 Bonn, Germany.; 2012;56(4):758–64. Available from: http://ovidsp.ovid.com/ovidweb.cgi?T=JS&PAGE=reference&D=medl&NEWS=N&AN=22173151

Nischalke H-D, Coenen M, Berger C, Aldenhoff K, Muller T, Berg T, et al. The toll-like receptor 2 (TLR2) -196 to -174 del/ins polymorphism affects viral loads and susceptibility to hepatocellular carcinoma in chronic hepatitis C. Int J Cancer [Internet]. United States: Nischalke,Hans-Dieter. Department of Internal Medicine I, University of Bonn, Bonn, Germany. nischalke@ukb.uni-bonn.de; 2012;130(6):1470–5. Available from: http://ovidsp.ovid.com/ovidweb.cgi?T=JS&PAGE=reference&D=medl&NEWS=N&AN=21500195

Okamoto K, Mimura K, Murawaki Y, Yuasa I. Association of functional gene polymorphisms of matrix metalloproteinase (MMP)-1, MMP-3 and MMP-9 with the progression of chronic liver disease. J Gastroenterol Hepatol [Internet]. Australia: Okamoto,Kinya. Division of Medicine and Clinical Science, Tottori University School of Medicine, Yonago, Japan.; 2005;20(7):1102–8. Available from: http://ovidsp.ovid.com/ovidweb.cgi?T=JS&PAGE=reference&D=med5&NEWS=N&AN=15955221

Pan Y, Zhao L, Chen X-M, Gu Y, Shen J-G, Liu L-M. The XRCC1 Arg399Gln genetic polymorphism contributes to hepatocellular carcinoma susceptibility: an updated meta-analysis. Asian Pac J Cancer Prev [Internet]. Thailand: Pan,Yan. Department of Integrative Oncology, Fudan University Shanghai Cancer Center, Shanghai, China E-mail : llm10010@126.com.; 2013;14(10):5761–7. Available from: http://ovidsp.ovid.com/ovidweb.cgi?T=JS&PAGE=reference&D=medl&NEWS=N&AN=24289575

Pasta L, Pietrosi G, Marrone C, D’Amico G, D’Amico M, Licata A, et al. C4BQ0: a genetic marker of familial HCV-related liver cirrhosis. Dig Liver Dis [Internet]. Netherlands: Pasta,L. Department of Medicine and Pneumology, V Cervello Hospital, Via Trabucco 180, 90146 Palermo, Italy. lindpas@yahoo.it; 2004;36(7):471–7. Available from: http://ovidsp.ovid.com/ovidweb.cgi?T=JS&PAGE=reference&D=med5&NEWS=N&AN=15285527

Patel K, Norris S, Lebeck L, Feng A, Clare M, Pianko S, et al. HLA class I allelic diversity and progression of fibrosis in patients with chronic hepatitis C. Hepatology [Internet]. United States: Patel,Keyur. Division of Gastroenterology, Duke Clinical Research Institute, Duke University Medical Center, Durham, NC 27715, USA.; 2006;43(2):241–9. Available from: http://ovidsp.ovid.com/ovidweb.cgi?T=JS&PAGE=reference&D=med5&NEWS=N&AN=16440356

Peng Q, Lao X, Chen Z, Lai H, Deng Y, Wang J, et al. TP53 and MDM2 gene polymorphisms, gene-gene interaction, and hepatocellular carcinoma risk: evidence from an updated meta-analysis. PLoS One [Internet]. United States: Peng,Qiliu. Department of Clinical Laboratory, First Affiliated Hospital of Guangxi Medical University, Nanning, Guangxi, China.; 2013;8(12):e82773. Available from: http://ovidsp.ovid.com/ovidweb.cgi?T=JS&PAGE=reference&D=medl&NEWS=N&AN=24376578

Petta S, Grimaudo S, Marco VD, Scazzone C, Macaluso FS, Camma C, et al. Association of vitamin D serum levels and its common genetic determinants, with severity of liver fibrosis in genotype 1 chronic hepatitis C patients. J Viral Hepat [Internet]. England: Petta,S. Cattedra di Gastroenterologia, DiBiMIS, University of Palermo, Palermo, Italy. petsa@inwind.it; 2013;20(7):486–93. Available from: http://ovidsp.ovid.com/ovidweb.cgi?T=JS&PAGE=reference&D=medl&NEWS=N&AN=23730842

Picelli N, Tanikawa AA, Grotto RMT, Silva GF, Barbosa AN, Ferrasi AC, et al. The absence of the human platelet antigen polymorphism effect on fibrosis progression in human immunodeficiency virus-1/hepatitis C virus coinfected patients. Rev Soc Bras Med Trop [Internet]. Brazil: Picelli,Natalia. Laboratorio de Biologia Molecular do Hemocentro, Faculdade de Medicina de Botucatu, Universidade Estadual Paulista Julio de Mesquita Filho, Botucatu, Sao Paulo, BR.; 2015;48(4):406–9. Available from: http://ovidsp.ovid.com/ovidweb.cgi?T=JS&PAGE=reference&D=medl&NEWS=N&AN=26312929

Plompen EPC, Darwish Murad S, Hansen BE, Loth DW, Schouten JNL, Taimr P, et al. Prothrombotic genetic risk factors are associated with an increased risk of liver fibrosis in the general population: The Rotterdam Study. J Hepatol [Internet]. 2015 Dec [cited 2016 Nov 3];63(6):1459–65. Available from: http://www.ncbi.nlm.nih.gov/pubmed/26226452

Powell EE, Edwards-Smith CJ, Hay JL, Clouston AD, Crawford DH, Shorthouse C, et al. Host genetic factors influence disease progression in chronic hepatitis C. Hepatology [Internet]. UNITED STATES: Powell,E E. Department of Gastroenterology and Hepatology, Princess Alexandra Hospital, University of Queensland, Brisbane, Australia.; 2000;31(4):828–33. Available from: http://ovidsp.ovid.com/ovidweb.cgi?T=JS&PAGE=reference&D=med4&NEWS=N&AN=10733535

Radwan MI, Pasha HF, Mohamed RH, Hussien HIM, El-Khshab MN. Influence of transforming growth factor-beta1 and tumor necrosis factor-alpha genes polymorphisms on the development of cirrhosis and hepatocellular carcinoma in chronic hepatitis C patients. Cytokine [Internet]. United States: Radwan,Mohamed I. Tropical Medicine Department, Faculty of Medicine, Zagazig University, Zagazig, Egypt.; 2012;60(1):271–6. Available from: http://ovidsp.ovid.com/ovidweb.cgi?T=JS&PAGE=reference&D=medl&NEWS=N&AN=22682513

Rahat B, Kiran M, Saxena R, Chawla YK, Sharma RR. Microsomal Epoxide Hydrolase Polymorphisms and Haplotypes as Determinants of Hepatitis B Virusand Hepatitis C Virus-related Liver Disease in Indian Population [Internet]. Journal of Clinical and Experimental Hepatology. J. Kaur, Department of Biochemistry, Institute of Medical Education and Research, Chandigarh - 160012, India. E-mail: jyotdeep2001@yahoo.co.in: Elsevier; 2012. p. 104–11. Available from: http://www.elsevier.com/wps/find/journaldescription.cws_home/725411/description#description

Real LM, Caruz A, Rivero-Juarez A, Soriano V, Neukam K, Rivero A, et al. A polymorphism linked to RRAS, SCAF1, IRF3 and BCL2L12 genes is associated with cirrhosis in hepatitis C virus carriers. Liver Int [Internet]. United States: Real,Luis M. Unidad de Enfermedades Infecciosas y Microbiologia, Hospital Universitario de Valme, Sevilla, Spain; Instituto de Biomedicina de Sevilla (IBIS), Sevilla, Spain.; 2014;34(4):558–66. Available from: http://ovidsp.ovid.com/ovidweb.cgi?T=JS&PAGE=reference&D=medl&NEWS=N&AN=24131527

Rizk NM, Derbala MF, N.M. R. Genetic polymorphisms of ICAM 1 and IL28 as predictors of liver fibrosis severity and viral clearance in hepatitis C genotype 4. Clin Res Hepatol Gastroenterol [Internet]. M.F. Derbala, Gastroenterology and Hepatology, Hamad Hospital, Doha, Qatar. E-mail: derbalamf@yahoo.com: Elsevier Masson SAS (62 rue Camille Desmoulins, Issy les Moulineaux Cedex 92442, France); 2013;37(3):262–8. Available from: http://ovidsp.ovid.com/ovidweb.cgi?T=JS&PAGE=reference&D=emed11&NEWS=N&AN=2013374512

Romero-Gomez M, Montes-Cano MA, Otero-Fernandez MA, Torres B, Sanchez-Munoz D, Aguilar F, et al. SLC11A1 promoter gene polymorphisms and fibrosis progression in chronic hepatitis C. Gut [Internet]. England: Romero-Gomez,M. Hepatology Unit, Hospital Universitario de Valme, Sevilla, Spain. mromerog@supercable.es; 2004;53(3):446–50. Available from: http://ovidsp.ovid.com/ovidweb.cgi?T=JS&PAGE=reference&D=med5&NEWS=N&AN=14960532

Rossi L, Leveri M, Gritti C, De Silvestri A, Zavaglia C, Sonzogni L, et al. Genetic polymorphisms of steroid hormone metabolizing enzymes and risk of liver cancer in hepatitis C-infected patients. J Hepatol [Internet]. England: Rossi,Laura. ASAEV, Via Garibaldi 13, 24040 Bonate Sotto (BG), Italy. laura.asaev@tiscalinet.it; 2003;39(4):564–70. Available from: http://ovidsp.ovid.com/ovidweb.cgi?T=JS&PAGE=reference&D=med4&NEWS=N&AN=12971967

Sato M, Kato N, Tateishi R, Muroyama R, Kowatari N, Li W, et al. IL28B minor allele is associated with a younger age of onset of hepatocellular carcinoma in patients with chronic hepatitis C virus infection. J Gastroenterol [Internet]. Japan: Sato,Masaya. Department of Gastroenterology, Graduate School of Medicine, The University of Tokyo, Tokyo, Japan.; 2014;49(4):748–54. Available from: http://ovidsp.ovid.com/ovidweb.cgi?T=JS&PAGE=reference&D=medl&NEWS=N&AN=23689989

Sato M, Kondo M, Tateishi R, Fujiwara N, Kato N, Yoshida H, et al. Impact of IL28B genetic variation on HCV-induced liver fibrosis, inflammation, and steatosis: a meta-analysis. PLoS One [Internet]. United States: Sato,Masaya. Department of Gastroenterology, Graduate School of Medicine, The University of Tokyo, Bunkyo-ku, Tokyo, Japan.; 2014;9(3):e91822. Available from: http://ovidsp.ovid.com/ovidweb.cgi?T=JS&PAGE=reference&D=medl&NEWS=N&AN=24637774

Sato S, Genda T, Hirano K, Tsuzura H, Narita Y, Kanemitsu Y, et al. Up-regulated aldo-keto reductase family 1 member B10 in chronic hepatitis C: association with serum alpha-fetoprotein and hepatocellular carcinoma. Liver Int [Internet]. United States: Sato,Shunsuke. Department of Gastroenterology and Hepatology, Juntendo University Shizuoka Hospital, Shizuoka, Japan.; 2012;32(9):1382–90. Available from: http://ovidsp.ovid.com/ovidweb.cgi?T=JS&PAGE=reference&D=medl&NEWS=N&AN=22681639

Scott BB, Egner W. Does alpha1-antitrypsin phenotype PiMZ increase the risk of fibrosis in liver disease due to hepatitis C virus infection?. Eur J Gastroenterol Hepatol [Internet]. England: Lippincott Williams and Wilkins; 2006;18(5):521–3. Available from: http://ovidsp.ovid.com/ovidweb.cgi?T=JS&PAGE=reference&D=med5&NEWS=N&AN=16607148

Segat L, Fabris A, Padovan L, Milanese M, Pirulli D, Lupo F, et al. MBL2 and MASP2 gene polymorphisms in patients with hepatocellular carcinoma. J Viral Hepat [Internet]. England: Segat,L. Genetic Service and Department of Developmental and Reproductive Sciences, IRCCS Burlo Garofoloand University of Trieste, Trieste, Italy. segat@burlo.trieste.it; 2008;15(5):387–91. Available from: http://ovidsp.ovid.com/ovidweb.cgi?T=JS&PAGE=reference&D=med5&NEWS=N&AN=18221301

Segat L, Milanese M, Pirulli D, Trevisiol C, Lupo F, Salizzoni M, et al. Secreted protein acidic and rich in cysteine (SPARC) gene polymorphism association with hepatocellular carcinoma in Italian patients. J Gastroenterol Hepatol [Internet]. Australia: Segat,Ludovica. Genetic Unit, IRCCS Burlo Garofolo and Department of Reproductive and Developmental Biology, University of Trieste, Trieste, Italy. segat@burlo.trieste.it; 2009;24(12):1840–6. Available from: http://ovidsp.ovid.com/ovidweb.cgi?T=JS&PAGE=reference&D=med5&NEWS=N&AN=19817957

Sermasathanasawadi R, Kato N, Muroyama R, Dharel N, Shao R-X, Chang J-H, et al. Association of interferon regulatory factor-7 gene polymorphism with liver cirrhosis in chronic hepatitis C patients. Liver Int [Internet]. England: Sermasathanasawadi,Radsamee. Department of Gastroenterology, Graduate School of Medicine, University of Tokyo, Tokyo, Japan.; 2008;28(6):798–806. Available from: http://ovidsp.ovid.com/ovidweb.cgi?T=JS&PAGE=reference&D=med5&NEWS=N&AN=18397234

Sertorio M, Hou X, Carmo RF, Dessein H, Cabantous S, Abdelwahed M, et al. IL-22 and IL-22 binding protein (IL-22BP) regulate fibrosis and cirrhosis in hepatitis C virus and schistosome infections. Hepatology [Internet]. United States: Sertorio,Mathieu. Aix-Marseille Universite, UMR_S 906, Marseille, France; Inserm, U906, Marseille, France.; 2015;61(4):1321–31. Available from: http://ovidsp.ovid.com/ovidweb.cgi?T=JS&PAGE=reference&D=medl&NEWS=N&AN=25476703

Shen X-F, Zeng X-T, Jian Z-Y, Zhou M, Zhou P. Quantitative assessment of the effect of epidermal growth factor 61A/G polymorphism on the risk of hepatocellular carcinoma [Internet]. Oncology Letters. Z.-Y. Jian, Department of General Surgery, Taihe Hospital, Hubei University of Medicine, 32 South Renmin Road, Shiyan, Hubei 442000, China: Spandidos Publications (10 Vriaxidos Street, Athens 116 10, Greece); 2015. p. 3199–205. Available from: http://www.spandidos-publications.com/ol/10/5/3199/download

Silva GF, Grotto RMT, Verdichio-Moraes CF, Corvino SM, Ferrasi AC, Silveira LV de A, et al. Human platelet antigen genotype is associated with progression of fibrosis in chronic hepatitis C. J Med Virol [Internet]. United States: Silva,Giovanni Faria. Gastroenterology Division, Department of Internal Medicine, Botucatu Medical School, Sao Paulo State University, UNESP, Botucatu-SP, Brazil.; 2012;84(1):56–60. Available from: http://ovidsp.ovid.com/ovidweb.cgi?T=JS&PAGE=reference&D=medl&NEWS=N&AN=22095535

Silvestri L, Sonzogni L, De Silvestri A, Gritti C, Foti L, Zavaglia C, et al. CYP enzyme polymorphisms and susceptibility to HCV-related chronic liver disease and liver cancer. Int J Cancer [Internet]. United States: Silvestri,Laura. Associazione Studi Avanzati Epatiti Virali, Bonate Sotto (BG), Italy.; 2003;104(3):310–7. Available from: http://ovidsp.ovid.com/ovidweb.cgi?T=JS&PAGE=reference&D=med4&NEWS=N&AN=12569554

Smith BC, Gorve J, Guzail MA, Day CP, Daly AK, Burt AD, et al. Heterozygosity for hereditary hemochromatosis is associated with more fibrosis in chronic hepatitis C. Hepatology [Internet]. UNITED STATES: Smith,B C. Centre for Liver Research, University of Newcastle upon Tyne, England, UK.; 1998;27(6):1695–9. Available from: http://ovidsp.ovid.com/ovidweb.cgi?T=JS&PAGE=reference&D=med4&NEWS=N&AN=9620344

Sonzogni L, Silvestri L, De Silvestri A, Gritti C, Foti L, Zavaglia C, et al. Polymorphisms of microsomal epoxide hydrolase gene and severity of HCV-related liver disease. Hepatology [Internet]. United States: Sonzogni,Laura. Associazione Studi Avanzati Epatiti Virali (ASAEV), Bonate Sotto (BG), Italy.; 2002;36(1):195–201. Available from: http://ovidsp.ovid.com/ovidweb.cgi?T=JS&PAGE=reference&D=med4&NEWS=N&AN=12085365

Stucker I, Loriot M-A, N’Koutchou G, Cenee S, Bodin L, Mulot C, et al. UDP-glucuronosyltransferase UGT1A7 genetic polymorphisms in hepatocellular carcinoma: A differential impact according to seropositivity of HBV or HCV markers? BMC Cancer [Internet]. England: Stucker,I. Inserm, U754, Villejuif, F-94807 France. stucker@vjf.inserm.fr; 2007;7:214. Available from: http://ovidsp.ovid.com/ovidweb.cgi?T=JS&PAGE=reference&D=med5&NEWS=N&AN=18021430

Suenaga M, Yamada S, Fujii T, Fuchs BC, Okumura N, Kanda M, et al. A functional polymorphism in the epidermal growth factor gene predicts hepatocellular carcinoma risk in Japanese hepatitis C patients [Internet]. OncoTargets and Therapy. S. Yamada, Department of Gastroenterological Surgery (Surgery II), Nagoya University Graduate School of Medicine, 65 Tsurumai-cho, Showa-ku, Nagoya 466-8550, Japan. E-mail: suguru@med.nagoya-u.ac.jp: Dove Medical Press Ltd. (PO Box 300-008, Albany, Auckland, New Zealand); 2013. p. 1805–12. Available from: http://www.dovepress.com/getfile.php?fileID=18456

Suneetha P V, Goyal A, Hissar SS, Sarin SK. Studies on TAQ1 polymorphism in the 3’untranslated region of IL-12P40 gene in HCV patients infected predominantly with genotype 3. J Med Virol [Internet]. United States: Suneetha,P V. Department of Gastroenterology, G.B. Pant Hospital, New Delhi, India.; 2006;78(8):1055–60. Available from: http://ovidsp.ovid.com/ovidweb.cgi?T=JS&PAGE=reference&D=med5&NEWS=N&AN=16789008

Suo GJ, Zhao ZX. Association of the interleukin-28B gene polymorphism with development of hepatitis virus-related hepatocellular carcinoma and liver cirrhosis: a meta-analysis. Genet Mol Res [Internet]. Brazil: Suo,G J. Department of Digestive Surgery, East Hospital, Tongji University School of Medicine, Shanghai, China guangjunsuo@hotmail.com.; 2013;12(3):3708–17. Available from: http://ovidsp.ovid.com/ovidweb.cgi?T=JS&PAGE=reference&D=medl&NEWS=N&AN=24085431

Takeuchi Y, Ikeda F, Moritou Y, Hagihara H, Yasunaka T, Kuwaki K, et al. The impact of patatin-like phospholipase domain-containing protein 3 polymorphism on hepatocellular carcinoma prognosis. J Gastroenterol [Internet]. Japan: Takeuchi,Yasuto. Department of Gastroenterology and Hepatology, Okayama University Graduate School of Medicine, Dentistry and Pharmaceutical Sciences, 2-5-1 Shikata-cho, Okayama, 700-8558, Japan.; 2013;48(3):405–12. Available from: http://ovidsp.ovid.com/ovidweb.cgi?T=JS&PAGE=reference&D=medl&NEWS=N&AN=22869157

Talaat RM, Esmail AA, Elwakil R, Gurgis AA, Nasr MI. Tumor necrosis factor-alpha -308G/A polymorphism and risk of hepatocellular carcinoma in hepatitis C virus-infected patients. Chin J Cancer [Internet]. China: Talaat,Roba M. Molecular Biology Department, Menofia University, Sadat, Egypt. Robamtalaat@yahoo.com; 2012;31(1):29–35. Available from: http://ovidsp.ovid.com/ovidweb.cgi?T=JS&PAGE=reference&D=medl&NEWS=N&AN=22200181

Tanaka Y, Furuta T, Suzuki S, Orito E, Yeo AET, Hirashima N, et al. Impact of interleukin-1beta genetic polymorphisms on the development of hepatitis C virus-related hepatocellular carcinoma in Japan. J Infect Dis [Internet]. United States: Tanaka,Yasuhito. Department of Clinical Molecular Informative Medicine, Nagoya City University Graduate School of Medical Sciences, Nagoya, Japan. ytanaka@med.nagoya-cu.ac.jp; 2003;187(11):1822–5. Available from: http://ovidsp.ovid.com/ovidweb.cgi?T=JS&PAGE=reference&D=med4&NEWS=N&AN=12751042

Tang K-S, Lee C-M, Teng H-C, Huang M-J, Huang C-S. UDP-glucuronosyltransferase 1A7 polymorphisms are associated with liver cirrhosis. Biochem Biophys Res Commun [Internet]. United States: Tang,Kung-Sheng. Department of Medical Technology, Fooyin University, Kaohsiung, Taiwan.; 2008;366(3):643–8. Available from: http://ovidsp.ovid.com/ovidweb.cgi?T=JS&PAGE=reference&D=med5&NEWS=N&AN=18054330

Tarhuni A, Guyot E, Rufat P, Sutton A, Bourcier V, Grando V, et al. Impact of cytokine gene variants on the prediction and prognosis of hepatocellular carcinoma in patients with cirrhosis. J Hepatol [Internet]. Netherlands: Tarhuni,Arige. INSERM, U773, Centre de Recherche Biomedicale, Bichat Beaujon CRB3, University Paris 7, Paris, France.; 2014;61(2):342–50. Available from: http://ovidsp.ovid.com/ovidweb.cgi?T=JS&PAGE=reference&D=medl&NEWS=N&AN=24751829

Tomoda T, Nouso K, Sakai A, Ouchida M, Kobayashi S, Miyahara K, et al. Genetic risk of hepatocellular carcinoma in patients with hepatitis C virus: a case control study. J Gastroenterol Hepatol [Internet]. Australia: Tomoda,Takeshi. Department of Gastroenterology and Hepatology, Okayama University Graduate School of Medicine, Dentistry, and Pharmaceutical Sciences, Okayama city, Japan. tomotake79@yahoo.co.jp; 2012;27(4):797–804. Available from: http://ovidsp.ovid.com/ovidweb.cgi?T=JS&PAGE=reference&D=medl&NEWS=N&AN=22004425

Trepo E, Nahon P, Bontempi G, Valenti L, Falleti E, Nischalke H-D, et al. Association between the PNPLA3 (rs738409 C>G) variant and hepatocellular carcinoma: Evidence from a meta-analysis of individual participant data. Hepatology [Internet]. United States: Trepo,Eric. Department of Gastroenterology, Hepatopancreatology and Digestive Oncology, Erasme Hospital, Universite Libre de Bruxelles, Brussels, Belgium; Laboratory of Experimental Gastroenterology, Universite Libre de Bruxelles, Brussels, Belgium.; 2014;59(6):2170–7. Available from: http://ovidsp.ovid.com/ovidweb.cgi?T=JS&PAGE=reference&D=medl&NEWS=N&AN=24114809

Trepo E, Pradat P, Potthoff A, Momozawa Y, Quertinmont E, Gustot T, et al. Impact of patatin-like phospholipase-3 (rs738409 C>G) polymorphism on fibrosis progression and steatosis in chronic hepatitis C. Hepatology [Internet]. United States: Trepo,Eric. Department of Gastroenterology, Hepatopancreatology, and Digestive Oncology, Erasme Hospital, Brussels, Belgium.; 2011;54(1):60–9. Available from: http://ovidsp.ovid.com/ovidweb.cgi?T=JS&PAGE=reference&D=med5&NEWS=N&AN=21488075

Tseng C-S, Tang K-S, Lo H-W, Ker C-G, Teng H-C, Huang C-S. UDP-glucuronosyltransferase 1A7 genetic polymorphisms are associated with hepatocellular carcinoma risk and onset age. Am J Gastroenterol [Internet]. United States: Tseng,Chien-Sen. Division of Hepatology, Center of Hepato-Gastroenterology, Yuan’s General Hospital, Kaohsiung, Taiwan.; 2005;100(8):1758–63. Available from: http://ovidsp.ovid.com/ovidweb.cgi?T=JS&PAGE=reference&D=med5&NEWS=N&AN=16086712

Tung BY, Emond MJ, Bronner MP, Raaka SD, Cotler SJ, Kowdley K V. Hepatitis C, iron status, and disease severity: relationship with HFE mutations. Gastroenterology [Internet]. United States: Tung,Bruce Y. Department of Medicine, University of Washington School of Medicine, Seattle, USA.; 2003;124(2):318–26. Available from: http://ovidsp.ovid.com/ovidweb.cgi?T=JS&PAGE=reference&D=med4&NEWS=N&AN=12557137

Turato C., Ruvoletto M.G., Biasiolo A., Quarta S., Tono N., Bernardinello E., et al. Squamous cell carcinoma antigen-1 (SERPINB3) polymorphism in chronic liver disease [Internet]. Digestive and Liver Disease. P. Pontisso, Department of Clinical and Experimental Medicine, University of Padua, Italy. E-mail: patrizia@unipd.it: Elsevier (P.O. Box 211, Amsterdam 1000 AE, Netherlands); 2009. p. 212–6. Available from: http://ovidsp.ovid.com/ovidweb.cgi?T=JS&PAGE=reference&D=emed9&NEWS=N&AN=2009088411

Urabe Y, Ochi H, Kato N, Kumar V, Takahashi A, Muroyama R, et al. A genome-wide association study of HCV-induced liver cirrhosis in the Japanese population identifies novel susceptibility loci at the MHC region. J Hepatol [Internet]. Netherlands: Urabe,Yuji. Laboratory of Molecular Medicine, Human Genome Center, Institute of Medical Science, The University of Tokyo, Tokyo, Japan.; 2013;58(5):875–82. Available from: http://ovidsp.ovid.com/ovidweb.cgi?T=JS&PAGE=reference&D=medl&NEWS=N&AN=23321320

Urbanek P, Lenicek M, Muchova L, Subhanova I, Dusek L, Kasprikova N, et al. No association of promoter variations of HMOX1 and UGT1A1 genes with liver injury in chronic hepatitis C. Ann Hepatol [Internet]. Mexico: Urbanek,Petr. Department of Internal Medicine, 1st. Faculty of Medicine, Charles University in Prague and Central Military Hospital, Prague, Czech Republic.; 2011;10(4):445–51. Available from: http://ovidsp.ovid.com/ovidweb.cgi?T=JS&PAGE=reference&D=med5&NEWS=N&AN=21911884

Valenti L, Pulixi E, Fracanzani AL, Dongiovanni P, Maggioni M, Orsatti A, et al. TNFalpha genotype affects TNFalpha release, insulin sensitivity and the severity of liver disease in HCV chronic hepatitis. J Hepatol [Internet]. England: Valenti,Luca. Dipartimento di Medicina Interna, Ospedale Maggiore IRCCS, Universita degli Studi, Pad Granelli, Via F Sforza 35, 20122 Milano, Italy.; 2005;43(6):944–50. Available from: http://ovidsp.ovid.com/ovidweb.cgi?T=JS&PAGE=reference&D=med5&NEWS=N&AN=16143422

Valenti L, Rumi M, Galmozzi E, Aghemo A, Del Menico B, De Nicola S, et al. Patatin-like phospholipase domain-containing 3 I148M polymorphism, steatosis, and liver damage in chronic hepatitis C. Hepatology [Internet]. United States: Valenti,Luca. Department of Internal Medicine, Universita degli Studi, Fondazione IRCCS Ospedale Maggiore Policlinico “Ca’ Granda” IRCCS, Milan, Italy. luca.valenti@unimi.it; 2011;53(3):791–9. Available from: http://ovidsp.ovid.com/ovidweb.cgi?T=JS&PAGE=reference&D=med5&NEWS=N&AN=21319195

Vogel A, Kneip S, Barut A, Ehmer U, Tukey RH, Manns MP, et al. Genetic link of hepatocellular carcinoma with polymorphisms of the UDP-glucuronosyltransferase UGT1A7 gene. Gastroenterology [Internet]. United States: Vogel,A. Department of Gastroenterology and Hepatology, Hannover Medical School, Hannover, Germany.; 2001;121(5):1136–44. Available from: http://ovidsp.ovid.com/ovidweb.cgi?T=JS&PAGE=reference&D=med4&NEWS=N&AN=11677206

Wang H, Mengsteab S, Tag C-G, Gao C-F, Hellerbrand C, Lammert F, et al. Transforming growth factor-beta1 gene polymorphisms are associated with progression of liver fibrosis in Caucasians with chronic hepatitis C infection. World J Gastroenterol [Internet]. China: Wang,Hao. Department of Laboratory Medicine, Changzheng Hospital, Second Military Medical University, Shanghai, China.; 2005;11(13):1929–36. Available from: http://ovidsp.ovid.com/ovidweb.cgi?T=JS&PAGE=reference&D=med5&NEWS=N&AN=15800982

Wang Y, Kato N, Hoshida Y, Otsuka M, Taniguchi H, Moriyama M, et al. UDP-glucuronosyltransferase 1A7 genetic polymorphisms are associated with hepatocellular carcinoma in japanese patients with hepatitis C virus infection. Clin Cancer Res [Internet]. United States: Wang,Yue. Department of Gastroenterology, Graduate School of Medicine, University of Tokyo, Tokyo, Japan.; 2004;10(7):2441–6. Available from: http://ovidsp.ovid.com/ovidweb.cgi?T=JS&PAGE=reference&D=med5&NEWS=N&AN=15073122

Wang Y, Kato N, Hoshida Y, Yoshida H, Taniguchi H, Goto T, et al. Interleukin-1beta gene polymorphisms associated with hepatocellular carcinoma in hepatitis C virus infection. Hepatology [Internet]. United States: Wang,Yue. Department of Gastroenterology, Graduate School of Medicine, University of Tokyo, Japan.; 2003;37(1):65–71. Available from: http://ovidsp.ovid.com/ovidweb.cgi?T=JS&PAGE=reference&D=med4&NEWS=N&AN=12500190

Wasmuth HE, Tag CG, Van de Leur E, Hellerbrand C, Mueller T, Berg T, et al. The Marburg I variant (G534E) of the factor VII-activating protease determines liver fibrosis in hepatitis C infection by reduced proteolysis of platelet-derived growth factor BB. Hepatology [Internet]. United States: Wasmuth,Hermann E. Department of Internal Medicine III, University Hospital Aachen, Aachen, Germany.; 2009;49(3):775–80. Available from: http://ovidsp.ovid.com/ovidweb.cgi?T=JS&PAGE=reference&D=med5&NEWS=N&AN=19105210

Weng C-J, Hsieh Y-H, Tsai C-M, Chu Y-H, Ueng K-C, Liu Y-F, et al. Relationship of insulin-like growth factors system gene polymorphisms with the susceptibility and pathological development of hepatocellular carcinoma. Ann Surg Oncol [Internet]. United States: Weng,Chia-Jui. Tainan University of Technology, Tainan, Taiwan.; 2010;17(7):1808–15. Available from: http://ovidsp.ovid.com/ovidweb.cgi?T=JS&PAGE=reference&D=med5&NEWS=N&AN=20119675

White D.L., Liu Y., Garcia J., El-Serag H.B., Jiao L., Tsavachidis S., et al. Sex hormone pathway gene polymorphisms are associated with risk of advanced hepatitis c-related liver disease in males [Internet]. International Journal of Molecular Epidemiology and Genetics. D.L. White, Section of Gastroenterology and Hepatology, Michael E. DeBakey VA Medical Center and Baylor College of Medicine, 2002 Holcombe Blvd (MS152), Houston, TX 77030, United States: E-Century Publishing Corporation (40 White Oaks Lane, Madison WI 53711, United States); 2014. p. 164–76. Available from: http://www.ijmeg.org/files/ijmeg0002411.pdf

Wright M, Goldin R, Hellier S, Knapp S, Frodsham A, Hennig B, et al. Factor V Leiden polymorphism and the rate of fibrosis development in chronic hepatitis C virus infection. Gut [Internet]. England: Wright,M. Hepatology Section, Division of Medicine A, Imperial College School of Medicine at St Mary’s Hospital, Praed St, London W2 1NY, UK. mark.wright@ic.ac.uk; 2003;52(8):1206–10. Available from: http://ovidsp.ovid.com/ovidweb.cgi?T=JS&PAGE=reference&D=med4&NEWS=N&AN=12865283

Yasui Y, Kudo A, Kurosaki M, Matsuda S, Muraoka M, Tamaki N, et al. Reduced organic anion transporter expression is a risk factor for hepatocellular carcinoma in chronic hepatitis C patients: a propensity score matching study. Oncology [Internet]. Switzerland: Yasui,Yutaka. Department of Gastroenterology and Hepatology, Musashino Red Cross Hospital, Tokyo, Japan.; 2014;86(1):53–62. Available from: http://ovidsp.ovid.com/ovidweb.cgi?T=JS&PAGE=reference&D=medl&NEWS=N&AN=24401597

Yee LJ, Tang J, Herrera J, Kaslow RA, van Leeuwen DJ. Tumor necrosis factor gene polymorphisms in patients with cirrhosis from chronic hepatitis C virus infection. Genes Immun [Internet]. England: Yee,L J. Program on the Epidemiology of Infection and Immunity, Department of Epidemiology and International Health, School of Public Health, University of Alabama at Birmingham, Birmingham, AL 35294-0007, USA.; 2000;1(6):386–90. Available from: http://ovidsp.ovid.com/ovidweb.cgi?T=JS&PAGE=reference&D=med4&NEWS=N&AN=11196686

Zekri A-RN, Salama H, Medhat E, Bahnassy AA, Morsy HM, Lotfy MM, et al. IL28B rs12979860 gene polymorphism in Egyptian patients with chronic liver disease infected with HCV. Asian Pac J Cancer Prev [Internet]. Thailand: Zekri,Abdel-Rahman N. Virology and Immunology Unit, Cancer Biology Department, Faculty of Medicine, National Cancer Institute, Cairo University, Cairo, Egypt E-mail : ncizekri@yahoo.com.; 2014;15(17):7213–8. Available from: http://ovidsp.ovid.com/ovidweb.cgi?T=JS&PAGE=reference&D=medl&NEWS=N&AN=25227816

Zhang C, Tian Y-P, Wang Y, Guo F-H, Qin J-F, Ni H. hTERT rs2736098 genetic variants and susceptibility of hepatocellular carcinoma in the Chinese population: a case-control study. Hepatobiliary Pancreat Dis Int [Internet]. China: Zhang,Chao. Tianjin Medical University General Hospital, Tianjin 300071, China.; 2013;12(1):74–9. Available from: http://ovidsp.ovid.com/ovidweb.cgi?T=JS&PAGE=reference&D=medl&NEWS=N&AN=23392802
