# Supplementary material for: Host genetic factors associated with hepatocellular carcinoma in patients with hepatitis C virus infection: A systematic review
Source: J Viral Hepat. 2018 Mar 1;25(5):442–56. doi: 10.1111/jvh.12871 (PMC6321980; doi:10.1111/jvh.12871)
Supplement: Supplementary file 3 [file JVH-25-442-s003.docx]

**APPENDIX C**:

This contains the full version of Tables 1 -4.

This (along with appendices A, B and D) can be accessed at

<https://figshare.com/s/2ffc9030826df2fe150e>
