# Supplementary material for: Host genetic factors associated with hepatocellular carcinoma in patients with hepatitis C virus infection: A systematic review
Source: J Viral Hepat. 2018 Mar 1;25(5):442–56. doi: 10.1111/jvh.12871 (PMC6321980; doi:10.1111/jvh.12871)
Supplement: Supplementary file 4 [file JVH-25-442-s004.docx]

# Appendix D: Search strategy

1. Hepatocellular carcinoma.tw.

2. HCC.tw.

3. Liver cancer.tw.

4. Liver carcinoma.tw.

5. Malig$ hepatoma.tw.

6. Liver neoplasm.tw.

7. Cirrho$.tw.

8. Severe fibrosis.tw.

9. End stage liver disease.tw.

10. 1 or 2 or 3 or 4 or 5 or 6 or 7 or 8 or 9

11. hepatitis c.tw.

12. HCV.tw.

13. 11 or 12

14. gene$.tw.

15. polymorph$.tw.

16. SNP.tw.

17. Single nucleotide polymorphism.tw.

18. GWAS.tw.

19. Genome wide association stud$.tw.

20. 14 or 15 or 16 or 17 or 18 or 19

21. Risk Factors/ or risk$.mp. or Risk/

22. Predict$.mp.

23. Associat$.tw.

24. 21 or 22 or 23

25. 10 and 13 and 20 and 24

26. review.pt.

27. 25 not 26

28. limit 27 to (English language and human)
